# Mild Photothermal Bimetallic Mesoporous Nanozyme Triggers Immunogenic Cell Death and Immune Contexture Remodeling for Precision Hepatocellular Carcinoma Treatment

**DOI:** 10.1002/advs.202512578

**Published:** 2025-09-25

**Authors:** Yanbing Huang, Lei Ding, Yanbing Cao, Qiang Feng, Yanjuan Li, Jianmin Wang, Zhixiong Cai, Fuli Xin, Peiyuan Wang, Jingfeng Liu

**Affiliations:** ^1^ Innovation Center for Cancer Research Clinical Oncology School of Fujian Medical University Fujian Cancer Hospital Fuzhou 350014 P. R. China; ^2^ Department of Hepatobiliary and Pancreatic Tumor Surgery Clinical Oncology School of Fujian Medical University Fujian Cancer Hospital Fuzhou 350014 P. R. China; ^3^ The United Innovation of Mengchao Hepatobiliary Technology Key Laboratory of Fujian Province Mengchao Hepatobiliary Hospital of Fujian Medical University Fuzhou 350007 P. R. China; ^4^ State Key Laboratory of Structure Chemistry Fujian Institute of Research on the Structure of Matter Chinese Academy of Sciences Fuzhou 350002 P. R. China; ^5^ Fujian Key Laboratory of Advanced Technology for Cancer Screening and Early Diagnosis Fujian Cancer Hospital Fuzhou 350014 P. R. China; ^6^ School of Rare Earths University of Science and Technology of China Hefei 230026 P. R. China

**Keywords:** bimetallic mesoporous nanozyme, hepatocellular carcinoma, immune contexture remodeling, immunogenic cell death, mild photothermal effect

## Abstract

Treating hepatocellular carcinoma (HCC) remains a significant clinical challenge because of its immunosuppressive tumor microenvironment (TME) and poor response to immunotherapy. Immunogenic cell death (ICD) has emerged as a promising strategy for enhancing antitumor immunity through the release of tumor‐associated antigens (TAAs) and damage‐associated molecular patterns (DAMPs). However, efficient ICD induction requires precise regulation of oxidative stress, which is hindered by the robust antioxidant defenses of tumors, particularly glutathione (GSH)‐mediated reactive oxygen species (ROS) scavenging. To address this, a platinum‐cobalt (Pt/Co) bimetallic nanozyme (BNzyme) with a mesoporous structure is developed, which exhibited enzymatic activities similar to those of peroxidase (POD) and glutathione peroxidase (GPx). The Pt/Co BNzyme not only catalyzed H_2_O_2_ to generate cytotoxic ROS but also simultaneously depleted GSH, amplifying oxidative stress and inducing tumor cell apoptosis. Additionally, near‐infrared laser irradiation enhanced its enzymatic activity through mild photothermal effects, further promoting ICD. In vivo and in vitro experiments demonstrated that the Pt/Co BNzyme effectively remodeled the immune TME by increasing TAAs and dendritic‐cell maturation. When combined with immune checkpoint inhibitors, this approach triggered robust systemic antitumor immunity and immune memory, effectively suppressing tumor growth and metastasis. The Pt/Co BNzyme exhibits excellent biosafety, offering a novel strategy for HCC immunotherapy.

## Graphical abstract

In this study, a Pt/Co BNzyme with peroxidase/GPx‐like activity is developed, increasing oxidative stress by generating ROS and depleting GSH to induce ICD. The mild PTT effect enhanced its enzymatic activity. The Pt/Co BNzyme remodeled the TME, promoting DC maturation. Combined with ICB, it triggered potent antitumor immunity and immune memory and suppressed tumor growth/metastasis with good biosafety, offering a promising HCC treatment strategy.

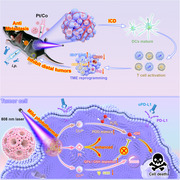

## 1 Introduction

The clinical treatment of hepatocellular carcinoma (HCC) faces difficult challenges, with persistently low survival rates closely linked to tumor immune evasion mechanisms.^[^ ^1^, ^2^ ^]^ Although T‐cell activation‐based immunotherapy has demonstrated revolutionary potential, its efficacy is severely compromised by the highly immunosuppressive tumor microenvironment (TME) and insufficient tumor immunogenicity.^[^ ^3^, ^4^ ^]^ Studies have revealed that the limited response to immunotherapy is attributed to three major barriers: 1) inadequate antigen presentation leading to weak immune activation signals;^[^ ^5^ ^]^ 2) abnormal accumulation of immunosuppressive cytokines and metabolites (e.g., interleukin‐10 (IL‐10), transforming growth factor‐β1 (TGF‐β1), and glutathione (GSH)) in the TME, impairing immune cell function;^[^ ^6^ ^]^ and 3) excessive immune checkpoint expression causing T‐cell exhaustion.^[^ ^7^ ^]^ In this context, immunogenic cell death (ICD) has become a key strategy for overcoming these limitations. ICD triggers the release of tumor‐associated antigens (TAAs), damage‐associated molecular patterns (DAMPs), and proinflammatory cytokines, thereby remodeling the immune microenvironment and activating systemic antitumor immunity.^[^ ^8^, ^9^ ^]^ However, efficient ICD induction critically depends on the precise regulation of tumor oxidative stress levels.

Recent studies have identified reactive oxygen species (ROS) as key factors in ICD initiation, yet their generation efficiency is constrained by the dynamic redox balance in the TME.^[^ ^10^, ^11^ ^]^ Tumor cells exploit high levels of GSH generation and other antioxidant defences to maintain an ROS‐scavenging capacity that far exceeds production rates, significantly decreasing the oxidative cytotoxicity of conventional therapies.^[^ ^12^ ^]^ Consequently, the development of nanocatalytic materials capable of simultaneously enhancing ROS generation and disrupting antioxidant defenses has become the focus of research.^[^ ^13^ ^]^ Many nanozymes have been demonstrated to function as natural catalyst mimics. The multienzyme catalytic properties (e.g., activities similar to those of peroxidase (POD), catalase (CAT) and glutathione peroxidase (GPx)) and environmental adaptability of metal‐ or metal oxide‐based nanozymes have garnered attention.^[^ ^14^, ^15^ ^]^ Single‐metal nanozymes (SNzymes) can amplify oxidative stress through cascade reactions that increase ROS levels while depleting GSH.^[^ ^16^ ^]^ However, their catalytic efficiency remains suboptimal for clinical applications, necessitating catalyst design innovations to optimise enzymatic performance.

Compared with SNzymes, bimetallic nanozymes (BNzymes) offer distinct advantages. The incorporation of multiple metals into the nanozyme structure enhances catalytic performance, stability, and versatility.^[^ ^17^, ^18^, ^19^ ^]^ Notably, the inherent high stability of multiple metal systems endows them with remarkable durability alongside their exceptional catalytic properties, rendering them highly effective in applications for cancer treatment.^[^ ^20^ ^]^ Platinum (Pt)‐based nanozymes have been widely studied for their exceptional enzyme‐like activity and stability.^[^ ^21^ ^]^ In addition, the photothermal effect of Pt is conducive to regulation of the enzyme reaction temperature, yet the scarcity and high cost of Pt hinder clinical translation.^[^ ^22^ ^]^ The synthesis of Pt‐based bimetallic nanomaterials using inexpensive metals is a reasonable strategy that can not only reduce the use of Pt but also improve catalytic performance.^[^ ^23^, ^24^ ^]^ Recent studies have suggested that Pt‐based bimetallic alloys can overcome the limitations of monometallic catalysts by leveraging ligand effects (electronic structure modulation) and strain effects (lattice contraction) to fine‐tune the d‐band occupancy of Pt surfaces, thereby optimising the adsorption strength of oxygen intermediates.^[^ ^25^, ^26^, ^27^ ^]^ Among these, platinum‐cobalt (Pt─Co) alloys exhibit superior oxygen reduction reaction activity and stability in the field of electrochemistry, offering a promising platform for developing potent ICD inducers.^[^ ^28^ ^]^

**Scheme 1:**
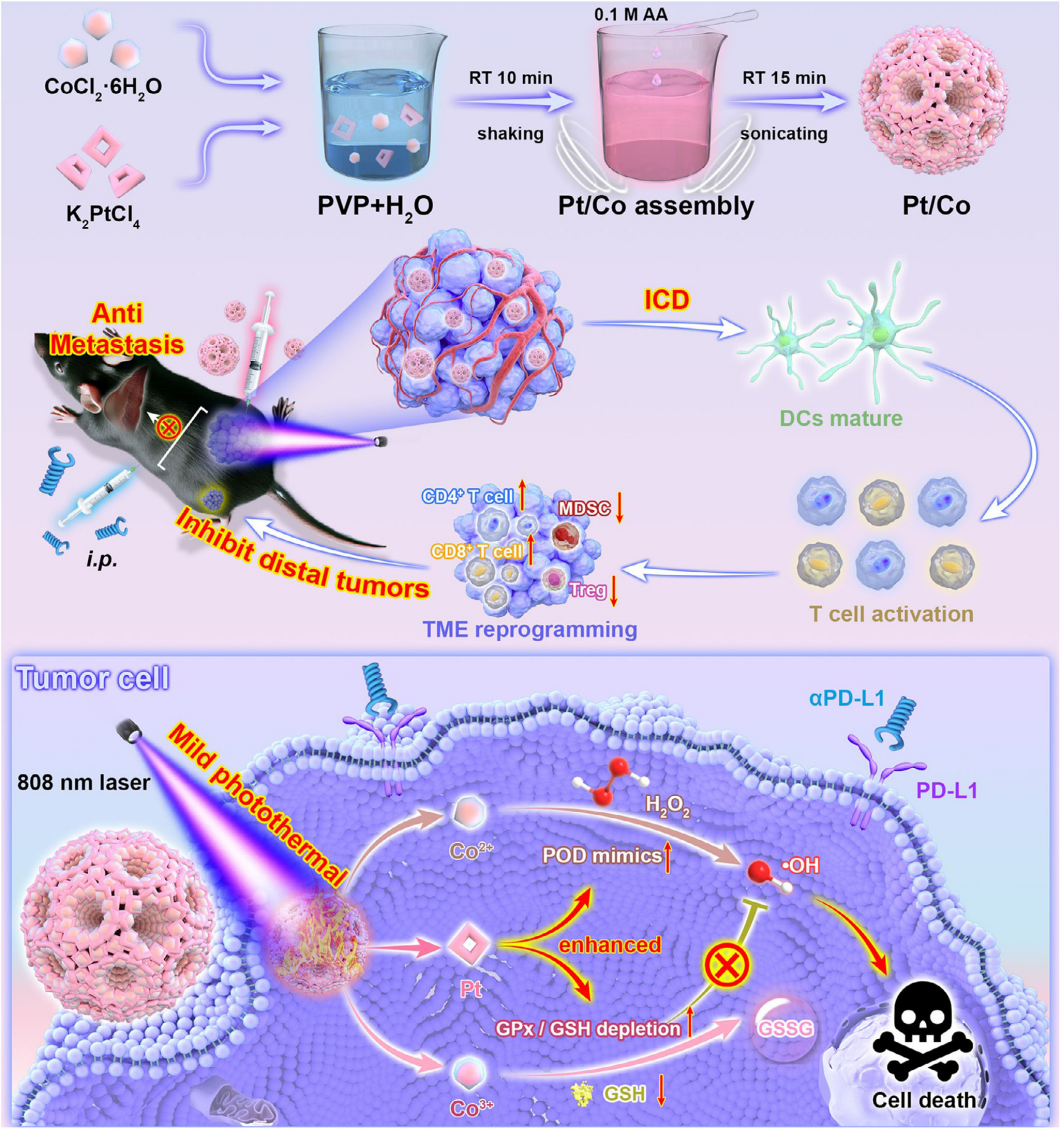
Synthesis and antitumor activity of the Pt/Co mesoporous BNzyme. The combination of enzymatic activity (POD and GPx), a mild photothermal effect, and an ICI could induce ICD, which promoted DC maturation, activated T cells, and reprogrammed the TME. This combination strategy not only inhibited tumor growth but also reduced lung metastasis.

Accordingly, following our previous research methodology,^[^ ^22^ ^]^ we reduced two types of metal precursors on preassembled polyvinylpyrrolidone (PVP) surfactant templates to construct a bimetallic Pt/Co mesoporous BNzyme. The mesoporous structure increases the reactive surface area and catalytic activity. The Pt/Co BNzyme exhibits POD‐like activity, leveraging hydrogen peroxide (H_2_O_2_) in the TME to generate ROS, thereby inducing tumor cell apoptosis. Moreover, Pt/Co demonstrate GPx‐like activity, catalysing the conversion of GSH to oxidised glutathione (GSSG), which reduces cellular antioxidant capacity and amplifies ROS‐mediated tumor cell damage through a cascade effect. Additionally, Pt exhibits excellent photothermal conversion capabilities.^[^ ^29^ ^]^ Under near‐infrared region (NIR) laser illumination, mild photothermal heating (42–45 °C) can achieve efficient therapy by disrupting thermoprotective mechanisms, catalysing the generation of reactive species, and modulating immune responses while markedly reducing tissue damage.^[^ ^30^, ^31^, ^32^ ^]^ Therefore, mild photothermal effects from Pt/Co further activate the enzymatic activities mentioned above, synergistically enhancing cell death.^[^ ^33^, ^34^ ^]^ Through this cascading and collaborative mode, the Pt/Co BNzyme effectively induces ICD in tumors, demonstrating robust antitumor efficacy both in vitro and in vivo. Moreover, this ICD can release abundant TAAs, promoting dendritic cell (DC) maturation and immune response activation. When combined with an immune checkpoint inhibitor (ICI), the Pt/Co BNzyme reshapes the immunosuppressive microenvironment: IL‐10 and TGF‐β1 levels and myeloid‐derived suppressor cell (MDSC) populations are reduced, while proinflammatory cytokine levels and cytotoxic T lymphocyte (CTL) infiltration are increased (**Scheme** **1**). This treatment also establishes immune memory, effectively suppressing lung metastasis. Compared with reported protocols for synthesizing bimetallic nanoenzymes for immunotherapy,^[^ ^35^, ^36^, ^37^ ^]^ our study presents a simple, green, one‐step method for synthesizing a Pt/Co bimetallic nanozyme. Through comprehensive, systematic evaluations, we demonstrate its ability to induce immune activation and durable immune memory in epidermal carcinoma, bilateral tumor, and multiple metastatic lesion models. Additionally, follow‐up transcriptomic (RNA‐seq) analysis corroborated its potent tumor‐suppressive efficacy, profoundly underscoring its potential for clinical HCC treatment.

## 2 Results and Discussion

### 2.1 Synthesis and Characterisation of the Pt/Co BNzyme

**Figure 1:**
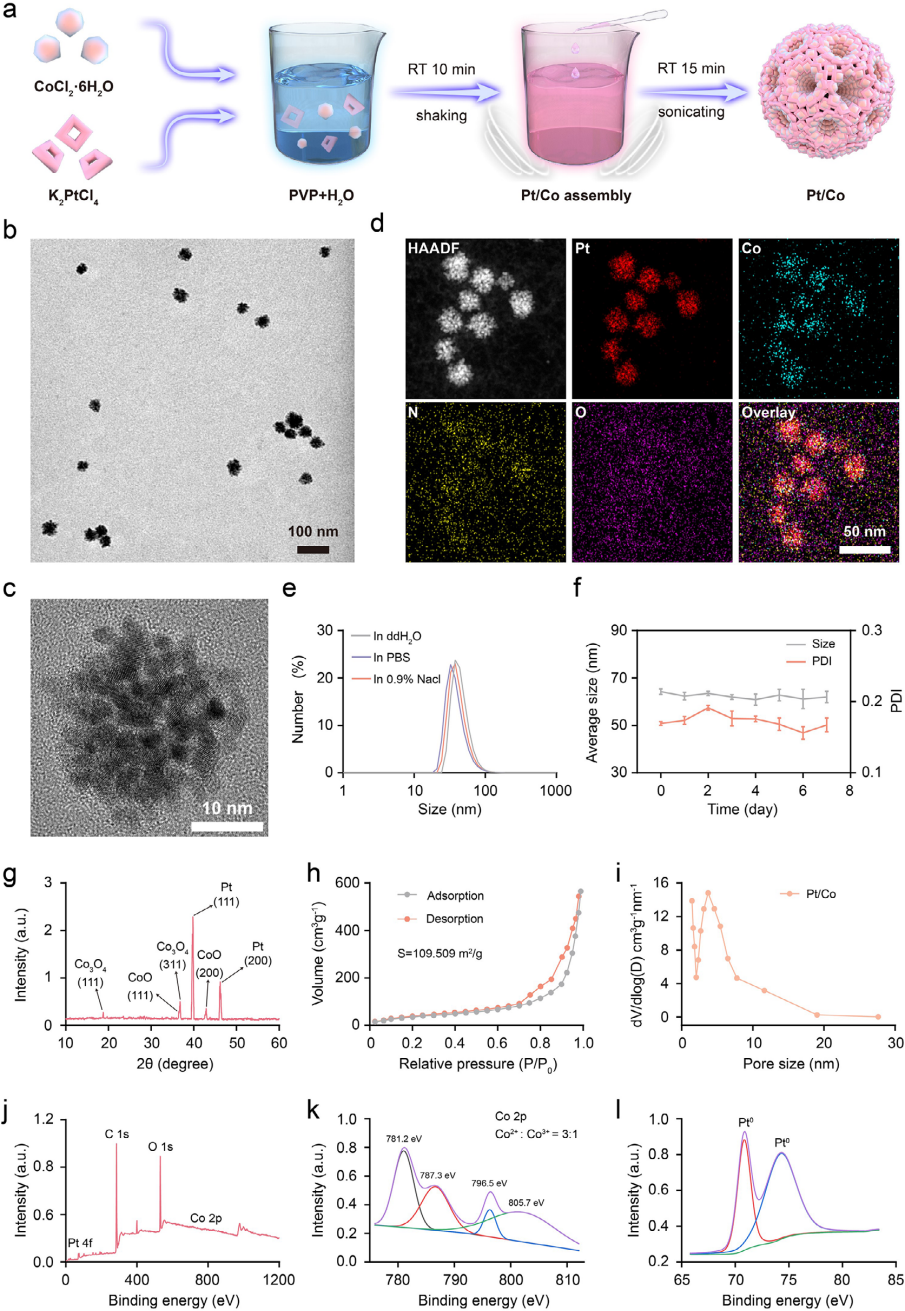
Synthesis and characterization of the Pt/Co mesoporous BNzyme. a) Pt and Co crystals gradually grew on the PVP template to form the Pt/Co BNzyme. b) TEM image and c) magnified TEM image of Pt/Co nanoparticles. Scale bar = 100 or 10 nm. d) HAADF‐STEM image of the Pt/Co BNzyme and corresponding elemental mapping images. Scale bar = 50 nm. e) DLS of the Pt/Co BNzyme dispersed in different solutions. f) Dynamic changes in the DLS metrics of the Pt/Co BNzyme over one week. The data are presented as the mean ± SD (n = 3). g) XRD spectrum of the Pt/Co BNzyme. h) N_2_ adsorption/desorption curves of the Pt/Co BNzyme. i) Pore size analysis of the Pt/Co BNzyme. Full XPS spectrum j), Co 2p spectrum k), and Pt 4f spectrum l) of the Pt/Co BNzyme by XPS.

In accordance with previous synthesis methods,^[^ ^22^ ^]^ the surfactant PVP served as both a capping ligand and a morphology‐directing agent. It coordinated to nascent particle surfaces through its carbonyl oxygen and lactam (pyrrolidone) ring, selectively passivating certain facets. The resulting facet‐dependent binding drove anisotropic growth as atoms were incorporated, steering the evolution toward branched, ramified architectures with inherent porosity.^[^ ^38^, ^39^ ^]^ Accordingly, both Pt and Co crystals gradually grew on the surfactant through a continuous reduction reaction, ultimately forming a bimetallic mesoporous nanozyme (**Figure** **1**a). Transmission electron microscopy (TEM) revealed that the Pt/Co BNzyme had a uniform dispersion and spherical morphology (Figure 1b,c). We also prepared Pt/Mn and Pt/Fe bimetallic alloy nanostructures. However, TEM (Figure S1, Supporting Information) showed that compared with Pt/Co, these two nanomaterials exhibited less distinct mesoporous structures, resulting in a smaller specific surface area. The elemental distribution of the Pt/Co BNzyme determined by high‐angle annular dark‐field scanning transmission electron microscopy (HAADF‐STEM, Figure 1d), revealed that Pt and Co were doped together and evenly dispersed in the spherical structure. Dynamic light scattering (DLS) data suggested that the average size of the Pt/Co BNzyme in ddH_2_O was ≈62 ± 2 nm (Figure 1e), and the diameter was similar in PBS and saline solution. The Pt/Co BNzyme was further dispersed in ddH_2_O, and the size and PDI did not significantly change over the course of one week, indicating good long‐term stability (Figure 1f). Notably, the zeta potential on the surface of the Pt/Co BNzyme was ‐11 ± 0.95 mV, which is beneficial for stability in blood circulation (Figure S2, Supporting Information). The crystal structure of the BNzyme was analysed by X‐ray diffraction (XRD). Pt species formed two sharp metal diffraction peaks at 39.8° (111) and 46.2° (200), indicating good crystallinity (Figure 1g). There were two species of Co crystals, leading to the formation of diffraction peaks for CoO species (at 36.5° (111) and 42.9° (200)) and Co_3_O_4_ species (at 18.8° (111) and 36.8° (311)). The mesoporous features of the Pt/Co BNzyme were detected by a nitrogen adsorption‒desorption trial (Figure 1h,i). A hysteresis loop appeared in the relative pressure (P/P_0_) range of 0.7–0.95, indicating a large, porous, specific surface area (S = 109.509 m^2^ g^−1^). In addition, the pore size of the BNzyme was ≈3.73 nm, which provides many active sites for enzyme reactions. Finally, X‐ray photoelectron spectroscopy (XPS) was employed to determine the composition of the elements and compounds in the Pt/Co BNzyme. The O 1s peak at 532 eV indicates the presence of oxide species in Pt/Co (Figure 1j). The signals at 781.2 and 796.5 eV were assigned to the Co 2p_3/2_ and Co 2p_1/2_ orbitals, respectively (Figure 1k). The ratio of Co^2+^ to Co^3+^ was ≈3:1. When a single Pt/Cu precursor was used, only mesoporous Pt nanoparticles were obtained (Figure S3, Supporting Information). Further, the lattice fringes of the porous Pt/Co were extended across the arms without obvious phase segregation, indicating the good mixing of Pt and Co (Figure S4, Supporting Information), Therefore, for Pt/Co fabrication, Pt precursors should be preferentially reduced to form Pt nuclei at the very initial stage owing to their high redox potential, then some extent of Co^2+^ was reduced with Co(Asc)_2_·xH_2_O was simultaneously incorporated into the Pt/Co alloy. Then, after thermally treatment under 200 °C for 12 h prior to bioapplication, both Co^2+^ and Co^3+^ were formed in the alloyed nanocatalyst. Co^2+^ could catalyze the generation of hydroxyl radicals (·OH) from high concentrations of H_2_O_2_, while Co^3+^ could oxidise GSH to GSSG.^[^ ^40^, ^41^ ^]^ Similarly, two peaks at 70.8 and 74.3 eV in the spectrum of Pt 4f were indexed to Pt^0^ (Figure 1l). Collectively, these findings demonstrate successful synthesis of the Pt/Co BNzyme and suggest the potential for multienzyme characteristics.

### 2.2 Enzymatic Activities and Photothermal Effects of the Pt/Co BNzyme

**Figure 2:**
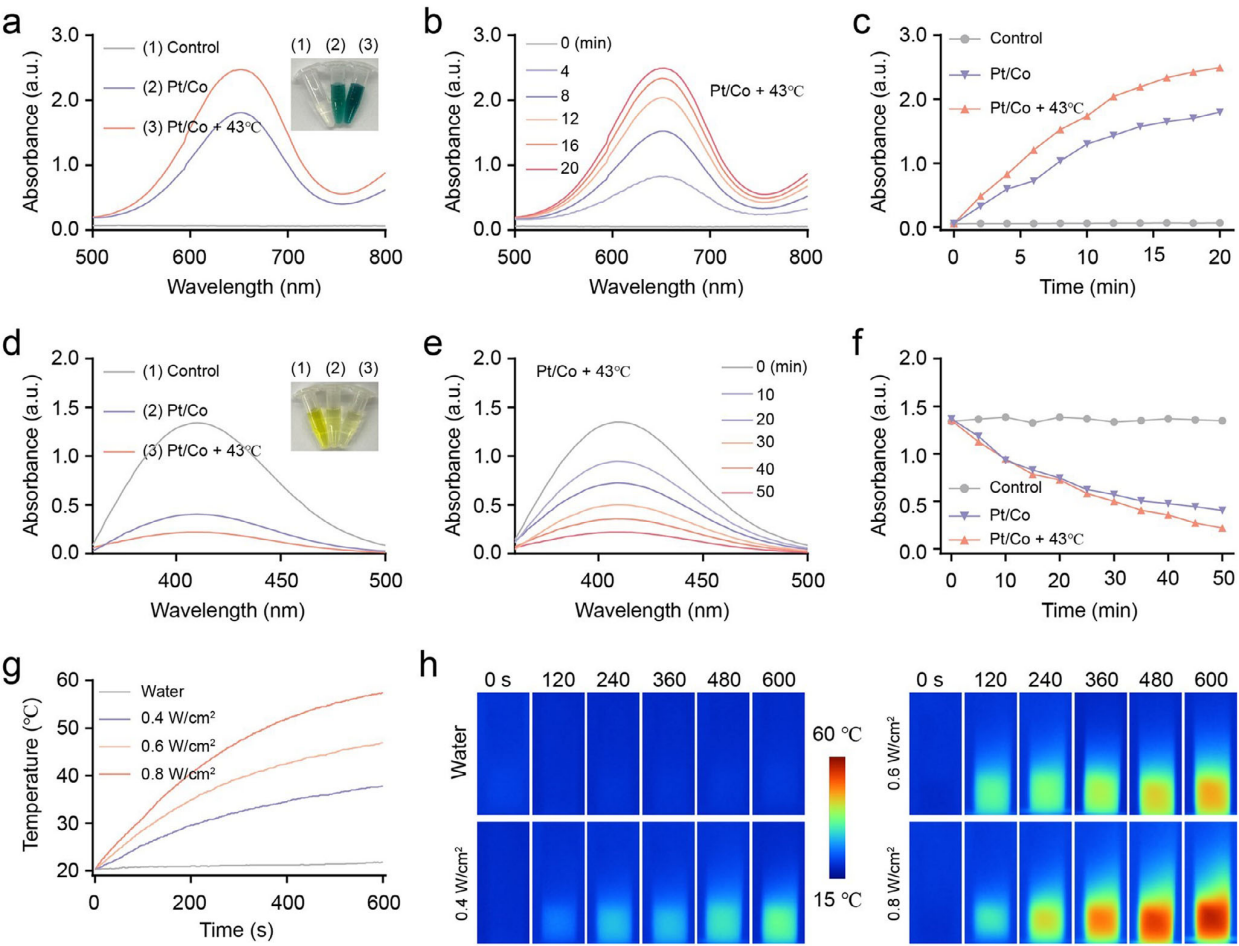
Enzymatic activities and photothermal effects of the Pt/Co BNzyme. a) UV–vis spectra and corresponding images of TMB + H_2_O_2_ solutions in various groups. b) Time‐dependent UV–vis spectra of the TMB + H_2_O_2_ solution treated with Pt/Co + 43 °C. c) The absorbance at 650 nm of the TMB + H_2_O_2_ solutions in the different groups. d) UV–vis absorption spectra and corresponding images of GSH consumption in the different groups detected by DTNB reagent. e) Time‐dependent absorption spectra of GSH consumption for Pt/Co + 43 °C detected by DTNB reagent. f) The absorbance at 412 nm of the DTNB reagent in the different groups. g) Changes in the temperature of the Pt/Co BNzyme solution irradiated by 808‐nm NIR light at different intensities. h) Infrared image of a Pt/Co BNzyme solution treated with different dosages of laser irritation; ddH_2_O was used as a control.

According to the composition of the Pt/Co BNzyme, we speculated that it was capable of enzymatic activities similar to those of POD and GPx. TMB solution (no color) can be oxidised into oxTMB (blue color) by OH. As shown in **Figure** **2**a, there was no color in TMB + H_2_O_2_ (5 mm) solution. However, when Pt/Co BNzyme was added to the solution, the color changed to blue. Under water bath heating (43 °C), the color became deeper, indicating that more oxidised TMB (oxTMB) was produced. In addition, (UV–vis) absorbance demonstrated that the POD‐like enzymatic activity was time‐dependent, with more OH being generated over time (Figure 2b; Figure S5, Supporting Information). The absorbance of oxTMB at 652 nm (OD_652_) indicated the ability of the Pt/Co BNzyme to produce OH (Figure 2c). In addition, mild temperatures accelerated this process and produced more·OH. Moreover, to further verify the POD‐like activity, we conducted electron paramagnetic resonance (EPR) testing, which further demonstrated that mild thermal treatment could significantly increase the enzyme‐like activity (Figure S6, Supporting Information). We also measured the OD_652_ after incubating Pt/Co with varying concentrations of TMB for various durations. From the calibration (standard) curve of OD_652_ versus time, we determined the slope (Figure S7, Supporting Information); simultaneously, by applying the Beer–Lambert law, a kinetic assay of Pt/Co with TMB was performed, and the results agreed well with those of the Michaelis–Menten model (Figure S8, Supporting Information). Meaningfully, the maximum initial velocity (V_max_) and Michaelis–Menten constant (K_m_) were fitted according to the Lineweaver–Burk double reciprocal curve. As illustrated in Figure S9 (Supporting Information), the K_m_ was calculated to be 0.97 mm, and the V_max_ was determined to be 1.94 × 10^−7^ M s^−1^; these values are significantly greater than those reported for an FePt‐based nanocatalyst,^[^ ^42^ ^]^ further indicating the satisfactory POD‐like activity of Pt/Co. The consumption of GSH was confirmed using Ellman's reagent (5,5′‐dithiobis‐(2‐nitrobenzoic acid), DTNB). DTNB, which is colorless, reacts with the free sulfhydryl groups of GSH to produce 2‐nitro‐5‐thiobenzoate (TNB), a yellow chromophore with a characteristic absorbance at 412 nm (OD_412_). In the control group, mixing 5 mm GSH with DTNB generated a strong yellow color (Figure 2d). In contrast, in the presence of Pt/Co BNzyme and H_2_O_2_, GSH was consumed via the GPx‐like catalytic cycle, thereby reducing the amount of free thiol available to react with DTNB, resulting in a diminished yellow intensity. Moreover, elevated temperature (43 °C) increased this activity such that more GSH was consumed. Similar to the POD‐like activity, the GPx‐like enzymatic activity was also time‐dependent (Figure 2e; Figure S10, Supporting Information). The OD_412_ results demonstrate the temperature‐ and time‐dependent GPx‐like enzymatic activity of the Pt/Co BNzyme (Figure 2f). Finally, the photothermal conversion effect of the Pt/Co BNzyme was verified. Under irradiation at 808 nm with an NIR laser, the temperature of the BNzyme solution increased significantly compared with that of the water solution (Figure 2g). With increasing irradiation time and increasing laser intensity, the temperature continued to increase (Figure 2h). To accurately determine the photothermal conversion efficiency (η), we tracked the temperature rise and decay of the Pt/Co system under repeated laser on/off cycles (Figure S11, Supporting Information). Using an established formula, η was determined to be ≈47.3% (Figure S12, Supporting Information), a value comparable to that reported for plasmonic Pt superstructures.^[^ ^43^ ^]^ According to these results, by controlling the intensity and duration of laser irradiation, we could obtain a mild temperature (43 °C) to achieve suitable enzyme reaction conditions for the Pt/Co BNzyme. This mesoporous BNzyme not only catalyzed H_2_O_2_ to generate cytotoxic ROS but also depleted GSH and induced mild thermal effects, which increased the oxidative state. Finally, we assessed the biodegradability of the Pt/Co BNzyme. The inability of a bimetal nanoenzyme to be effectively degraded in vivo could result in accumulation over time, potentially causing toxic or immune responses. TEM revealed that the Pt/Cu BNzyme gradually disintegrated in the tumor microenvironment with high H_2_O_2_ concentrations and was fully degraded within 24 h (Figure S13, Supporting Information). Consequently, the Pt/Co alloy nanocatalyst we developed not only showed exceptional tumor‐specific enzyme‐like capabilities but also demonstrated high biosafety, positioning it as a promising candidate for clinical translation as a nanomedicine.

### 2.3 Antitumor Effects of the Pt/Co BNzyme In Vitro

**Figure 3:**
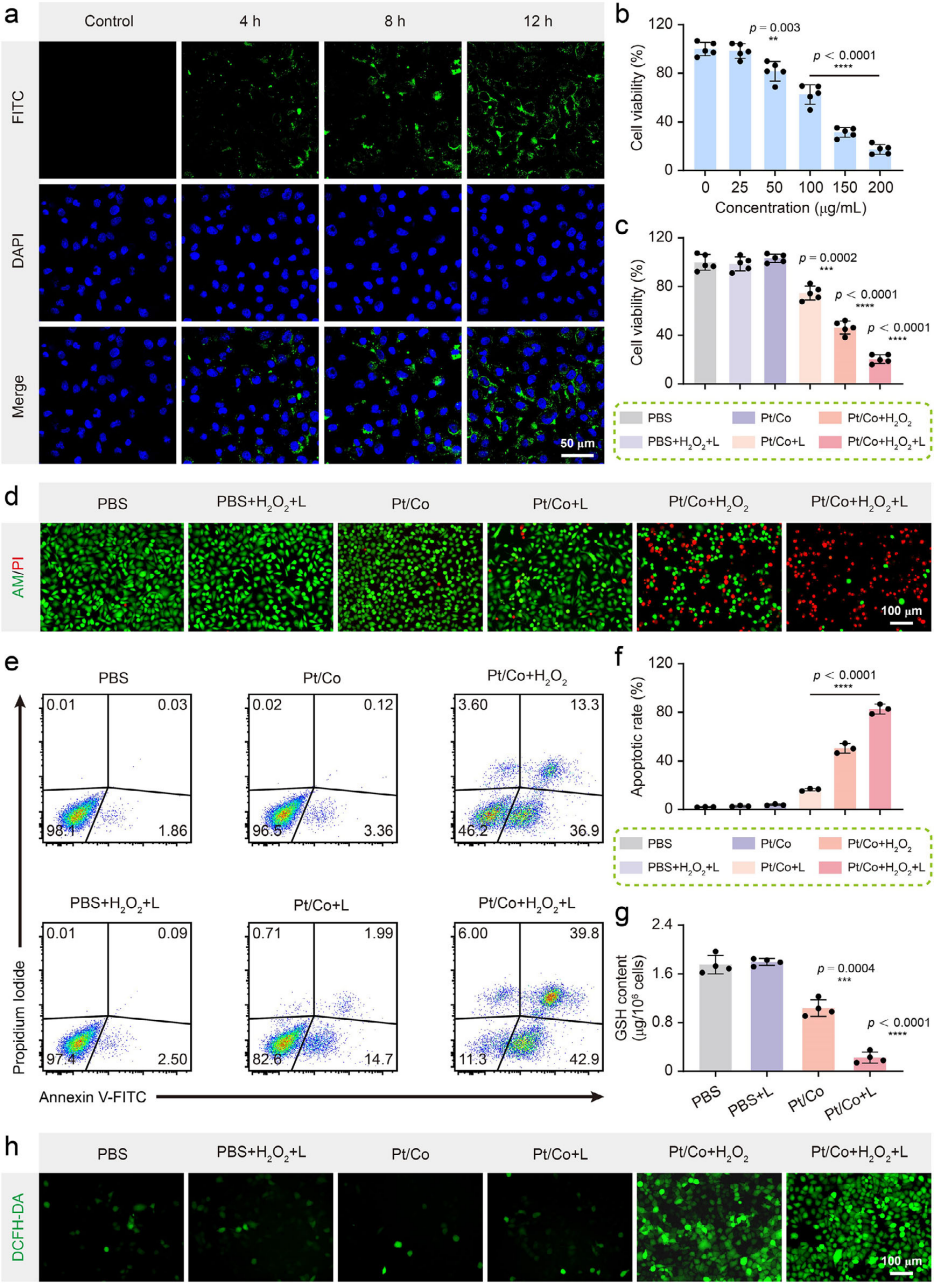
Antitumor effects of the Pt/Co BNzyme in vitro. a) CLSM images of the Pt/Co BNzyme incubated with Hepa1‐6 cells for different durations. Scale bar = 50 µm. b) Viability of Hepa1‐6 cells incubated with different concentrations of the Pt/Co BNzyme under H_2_O_2_ and laser irradiation conditions (n = 5). c) Viability of Hepa1‐6 cells subjected to different treatments detected by CCK‐8 assay (n = 5). d) Viability of Hepa1‐6 cells subjected to different treatments detected by AM/PI staining. Scale bar = 100 µm. e) The apoptotic state of Hepa1‐6 cells subjected to different treatments detected by Annexin‐V/PI staining. f) Average apoptotic rates in the different treatment groups shown in Figure 3e (n = 3). g) Intracellular GSH content of Hepa1‐6 cells subjected to different treatments detected by DTNB reagent (n = 4). h) Intracellular ROS levels in Hepa1‐6 cells subjected to different treatments detected by DCFH‐DA reagent. Scale bar = 100 µm. The data are presented as the mean ± SD (^**^ *p* < 0.01, ^***^ *p* < 0.001, and ^****^ *p* < 0.0001).

On the basis of the properties of the BNzyme described above, we proceeded with antitumor experiments in vitro. Different concentrations of Pt/Co BNzyme were incubated with tumor (Hepa1‐6) and normal (BNL CL.2) cells. Even at high concentrations (400 µg mL^−1^), nearly 92.3% of the cells remained alive, indicating the low inherent cytotoxicity of the BNzyme (Figure S14, Supporting Information). Encouragingly, in BNL CL.2 cells, we detected only trace levels of ROS generation after coculture with Pt/Co even under laser irradiation (Figure S15, Supporting Information), further confirming its excellent biosafety profile. The uptake of FITC‐labeled Pt/Co BNzyme by Hepa1‐6 cells was observed by confocal laser scanning microscopy (CLSM). As time progressed, more green fluorescence appeared in the tumor cells (**Figure** **3**a; Figure S16, Supporting Information). These cells were subsequently digested and analysed by flow cytometry. These results were consistent with the CLSM results, indicating that BNzyme was effectively internalized by these cells (Figure S17, Supporting Information). The ability of the Pt/Co BNzyme to inhibit tumor growth was tested by CCK‐8 assay (Figure 3b). Pt/Co BNzyme or H_2_O_2_ alone did not harm tumor cells. However, with the addition of H_2_O_2_, the Pt/Co BNzyme exhibited POD‐ and GPx‐like enzymatic activity, inducing significant antitumor effects. In addition, the Pt/Co BNzyme had a photothermal effect; under mild heating, the enzymatic activity increased, resulting in increased tumor cell death (Figure 3b). The combination of the enzymatic activity with the photothermal effect significantly inhibited cell growth, and the cell viability decreased as the amount of Pt/Co BNzyme concentration increased (Figure 3c). Similar results were also obtained by live/dead staining (Figure 3d; Figure S18, Supporting Information). The fluorescence of calcein‐AM/propidium iodide (PI) revealed that nearly half of the tumor cells were killed because of enzymatic activity (Pt/Co + H_2_O_2_ group). Under mild heating induced by NIR laser irradiation, the killing effect was significantly enhanced, resulting in the death of nearly all tumor cells (Pt/Co + H_2_O_2_ + L group). Notably, most cells died by apoptosis (Figure 3e; Figure S19, Supporting Information). Annexin‐V staining, indicating apoptosis, revealed the death of nearly all cells. Mild photothermal heating significantly promoted enzymatic activity, which led to an apoptosis rate of more than 82.7 ± 4.1% (Figure 3f). Consistently, caspase‐3/7 luminescence assays revealed a progressive increase in enzymatic activity across treatment groups, with Pt/Co + H_2_O_2_ + L inducing the most pronounced activation (Figure S20, Supporting Information). This strong activation of caspase cascades further confirmed that cell death was predominantly mediated by apoptosis rather than necrosis. To explore the mechanism of death, we detected the intracellular ROS and GSH contents using DCFH‐DA and DTNB. Owing to the large amount of·OH produced by the POD‐like enzymatic effect, more green fluorescence was detected in the Pt/Co + H_2_O_2_ group (Figure 3h). The JC‐1 probe is widely utilised to detect mitochondrial damage; in healthy mitochondria, JC‐1 forms red J‐aggregates, whereas in damaged mitochondria, it remains in its green monomeric form. Remarkably, treatment with Pt/Co + L and Pt/Co + H_2_O_2_ led to the loss of red fluorescence and an increase in green fluorescence (Figure S21a, Supporting Information). Importantly, the green fluorescence in the Pt/Co + H_2_O_2_ + L group was significantly elevated, indicating the most severe mitochondrial dysfunction, with the mitochondrial membrane potential (ΔΨm) decreasing by 1.8‐fold compared with that in the Pt/Co + H_2_O_2_ group (Figure S21b, Supporting Information). Therefore, these ROS can cause changes in calcium ion levels and mitochondrial function to induce apoptosis.^[^ ^44^, ^45^ ^]^ Moreover, the GPx‐like enzymatic effect resulted in the consumption of intracellular GSH, which impaired the antioxidant capacity of tumor cells, contributing to the increase in the·OH level (Figure 3g). In the same manner, the mild temperature increase reinforced these two enzymatic effects.

**Figure 4:**
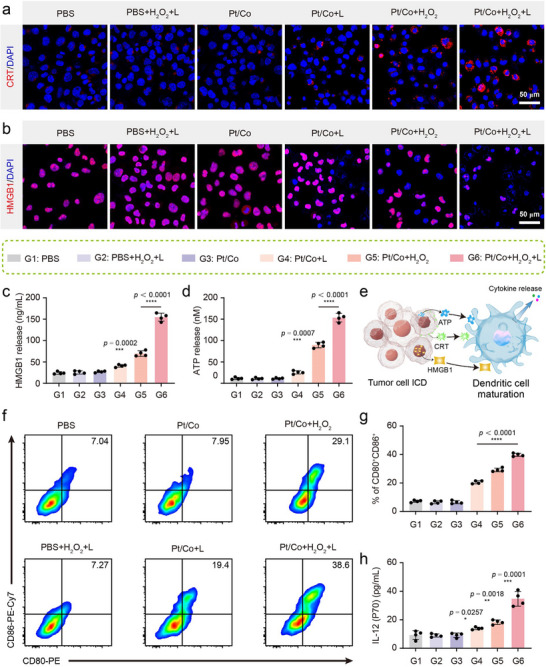
Pt/Co BNzyme induced ICD and immune responses in vitro. Immunofluorescence images of CRT a) and HMGB1 b) expression in Hepa1‐6 cells subjected to various treatments. Scale bar = 50 µm. The amounts of HMGB1 c) and ATP d) released into the supernatant of Hepa1‐6 cell cultures subjected to different treatments. e) ICD of tumor cells could promote DC maturation, inducing cytokine release. f) DCs and Hepa1‐6 tumor cells were incubated in a Transwell coculture system and subjected to different treatments, and the maturation of CD80^+^CD86^+^ DCs was detected by flow cytometry. g) Average maturation rates of DCs in the different treatment groups shown in Figure 4f. h) The content of secreted IL‐12 in the supernatant shown in Figure 4f, as determined by ELISA. The data are expressed as the mean ± SD (n = 4), with significance levels indicated as follows: ^*^ *p* < 0.05, ^**^ *p* < 0.01, ^***^ *p* < 0.001, and ^****^ *p* < 0.0001.

ICD is considered a critical initiating factor in the immune response.^[^ ^46^ ^]^ When ICD occurs, more calreticulin (CRT) is expressed and translocated to the outer side of the cell membrane; at the same time, high‐mobility group box‐1 protein (HMGB1) and adenosine triphosphate (ATP) are released from the cell.^[^ ^47^, ^48^ ^]^ To verify the type of tumor death induced by treatment with the Pt/Co BNzyme, Hepa1‐6 cells subjected to different treatments were subjected to immunofluorescence (Figures S22 and S23, Supporting Information). More CRT was clearly present in the Pt/Co + H_2_O_2_ + L group (**Figure** **4**a). As a result of the release of HMGB1, less fluorescence was detected in the combined group (Figure 4b). The supernatant of the tumor cell cultures was subsequently collected and tested by ELISA. Consistent with the fluorescence results, the content of HMGB1 was prominently elevated in the supernatant of the Pt/Co + H_2_O_2_ + L group (Figure 4c). In addition, the ATP content was also increased in the supernatant (Figure 4d). Encouragingly, we discovered that the HMGB1, CRT and APT levels in the Pt/Co+H_2_O_2_+L group were nearly comparable to those in the oxaliplatin (OXA)‐treated positive control group (Figure S24, Supporting Information). These results demonstrate that the Pt/Co BNzyme induced ICD and that a mild temperature increase enhanced this phenomenon. As shown in Figure 4e, increased amounts of DAMPs could be recognized by DCs, prompting the DCs to mature and release cytokines, such as IL‐12 and tumor necrosis factor alpha (TNF‐α). To verify this mechanism in vitro, a Transwell chamber model was established in which tumor cells were added to the upper chamber and DCs were added to the lower chamber. Tumor cells were subjected to different pretreatments and then cocultured with DCs using a Transwell system for 48 h without direct cell contact. Then, the DCs were digested and analysed by flow cytometry (Figure 4f; Figure S25, Supporting Information). Mature DCs were characterized by the expression of CD80^+^CD86^+^ on the cell membrane. Compared with that in the PBS group, the percentage of mature DCs was more than 39.8 ± 1.1% greater in Pt/Co + H_2_O_2_ + L group (Figure 4g). Simultaneously, the cytokine content in the supernatant was detected by ELISA. The levels of both IL‐12 (Figure 4h) and TNF‐α (Figure S26, Supporting Information) secreted by mature DCs were significantly increased in the Pt/Co + H_2_O_2_ + L group. These results demonstrate that the combination of enzymatic activity and photothermal effects induced ICD and DC maturation, which encouraged us to conduct subsequent in vivo experiments.

### 2.4 Antitumor Effects of the Pt/Co BNzyme In Vivo

Before the BNzyme was applied in mice, we first carried out a biosafety test. Different concentrations of Pt/Co BNzyme were mixed with the same amount of erythrocyte suspension extracted from C57BL/6 mouse blood. As shown in Figure S27 (Supporting Information), all erythrocytes swelled in ddH_2_O, while fewer than 5% of the blood cells were destroyed in the BNzyme solution, indicating good blood compatibility. Then, 5 mg kg^−1^ Pt/Co was injected through the tail vein into healthy C57BL/6 mice to observe safety in vivo. Compared with those in mice injected with PBS, major biochemical and blood cell indicators were not significantly different in those injected with BNzyme, indicating that Pt/Co did not affect the homeostasis of the body (Figure S28, Supporting Information).

**Figure 5:**
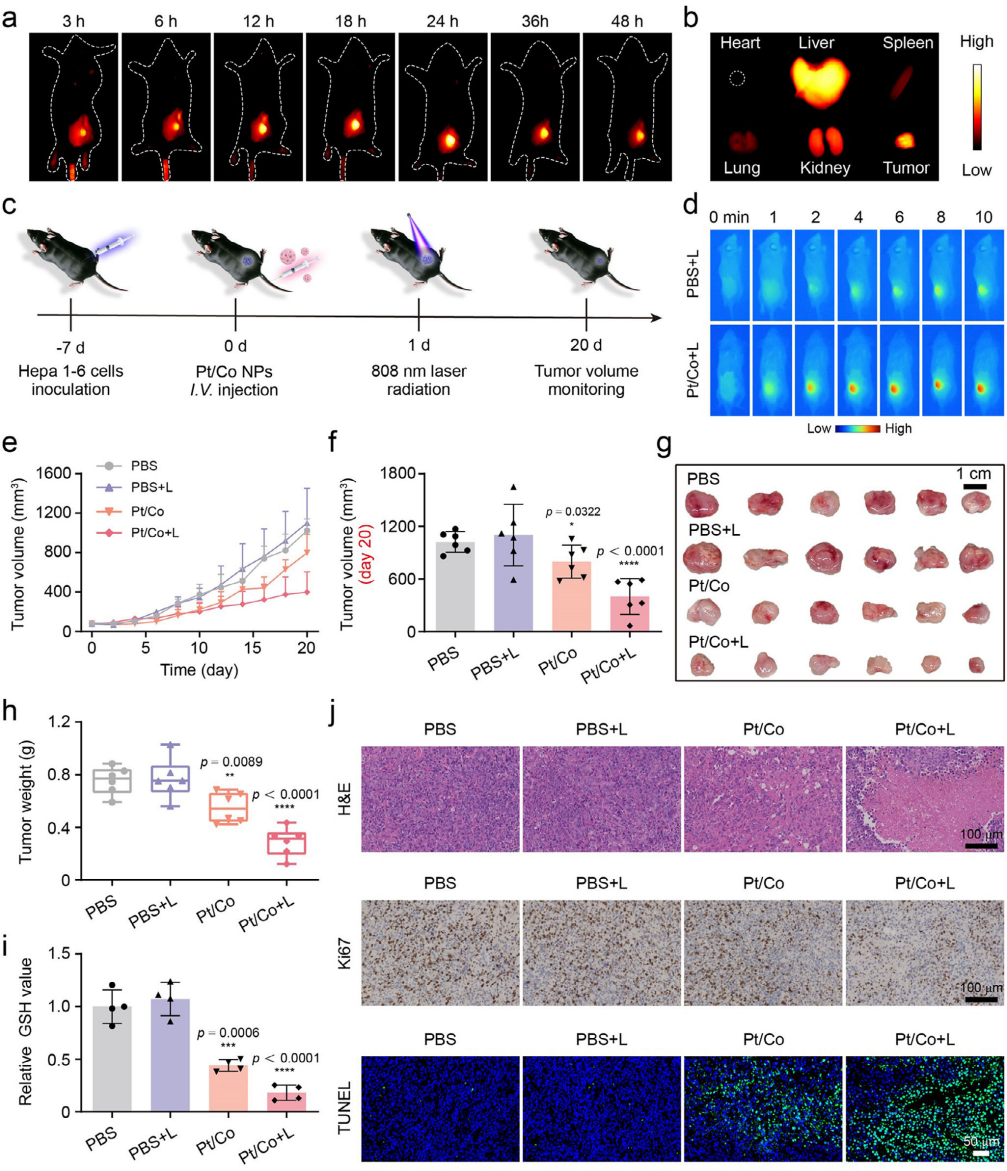
Antitumor effects of the Pt/Co BNzyme in vivo. a) Fluorescence images of Pt/Co BNzyme (ICG‐labeled) enrichment in Hepa1‐6 tumor‐bearing C57BL/6 mice. b) *Ex vivo* fluorescence images of tumors and major organs at 48 h corresponding to Figure 5a. c) Flow chart of antitumor experiments in vivo. d) Infrared images of Hepa1‐6 tumors irradiated by an NIR laser after different treatments. e) Changes in tumor volume in mice subjected to different treatments. f) Average tumor volume on day 20 after different treatments. g) *Ex vivo* images of tumors on day 20 after different treatments. Scale bar = 1 cm. h) Average tumor weight on day 20 after different treatments. i) Comparison of the relative GSH content in tumor tissue homogenates from the different treatment groups and the PBS group. j) H&E‐, Ki67‐ and TUNEL‐stained tumor tissues subjected to different treatments. Scale bar = 50 or 100 µm. The data are presented as the mean ± SD (n = 4) (^*^ *p* < 0.05, ^**^ *p* < 0.01, ^***^ *p* < 0.001, and ^****^ *p* < 0.0001).

Owing to the EPR effect, nanozymes are more likely to penetrate tumor tissue and remain for a long time.^[^ ^49^, ^50^ ^]^ We labeled the BNzyme with ICG for the real‐time monitoring of in vivo tumor accumulation. Due to the confined packing of mesopores, coordination with Co ions, and formation of J‐aggregates under NIR irradiation, ICG leakage was effectively prevented. Because of the photobleaching of ICG in aqueous solution, the photostability of ICG and ICG‐labeled Pt/Co in water was subsequently measured using a fluorescence bioimaging system. Free ICG lost ≈93.2% of its initial fluorescence intensity after continuous illumination for 25 min. In contrast, ICG‐labeled Pt/Co retained almost ≈92.1% of the initial signal intensity at the end of continuous irradiation (Figures S29 and S30), demonstrating that the mesoporous nanozyme could be further used as a nanocarrier for ICG delivery. Accordingly, after being injected through the tail vein, ICG‐labeled Pt/Co BNzyme showed enrichment in Hepa1‐6 tumors in C57BL/6 mice, with brighter fluorescence than that observed in other areas (**Figure** **5**a). At 24 h, the tumor showed the greatest fluorescence intensity, indicating the greatest BNzyme enrichment at this time (Figure S31, Supporting Information). Then, the main organs of the treated mice were collected to observe the biological distribution of the BNzyme. Nanomaterials, when injected in vivo, are very susceptible to being captured by the reticuloendothelial system.^[^ ^51^ ^]^ Accordingly, we detected the presence of Pt/Co in the liver; however, greater nanoenzyme and fluorescence levels were observed in the tumor tissue (Figure 5b; Figure S32, Supporting Information). In addition, to further validate the tumor‐targeting capability of Pt/Co, the Pt contents in the tumor and vital organs were evaluated by inductively coupled plasma‒optical emission spectrometry (ICP‐OES). The highest content of Pt was found in the tumor tissue at 24 h after injection (Figure S33, Supporting Information), which was consistent with the fluorescence imaging results (Figure 5a). Moreover, the Pt levels in organs declined rapidly over time after injection; by day 7, only trace amounts remained (Figure S33, Supporting Information), further demonstrating excellent biosafety. Notably, by measuring the pharmacokinetics of Pt/Co, we determined that it has a blood circulation half‐life of 7.84 h (Figure S34, Supporting Information), which is much longer than that of Au nanocrystals of similar size^[^ ^52^ ^]^ and is primarily attributable to the mesoporous nanostructure.

Next, the antitumor effect of the Pt/Co BNzyme was investigated in vivo (Figure 5c). When the tumor volume reached 80–100 mm^3^, the mice were divided into four groups: the PBS, PBS +L, Pt/Co, and Pt/Co + L groups. According to the above enrichment results, we carried out photothermal treatment at 24 h after injection. Under laser irradiation at 808 nm (0.6 W cm^−2^, 10 min), the tumor temperature in the Pt/Co + L group increased to 42.7 °C within 4 min, reaching a final temperature of 44.8 °C, while the temperature in the PBS + L group did not exceed 38 °C, indicating a certain thermal effect of Pt/Co + L (Figure 5d; Figure S35, Supporting Information). The mild temperature increase induced by the photothermal effect could promote both enzymatic and immune activity, thereby synergistically enhancing the killing of tumor cells.^[^ ^53^, ^54^ ^]^ During the treatment period, the tumor volume (Figure 5e; Figure S35, Supporting Information) and body weight (Figure S37, Supporting Information) of the mice were measured every 2 days. The tumor volume was similar in the PBS and PBS+L groups. Owing to the high levels of GSH and H_2_O_2_ in the TME, the POD‐ and GPx‐like enzymatic activities of Pt/Co BNzyme efficiently inhibited tumor growth compared with that observed in the control group. With the assistance of a mild temperature increase, the enzymatic activities in the Pt/Co + L group were markedly greater than those in the Pt/Co group (*p* < 0.0001; Figure 5f; Figure S38, Supporting Information). At the end of the observation period, all tumors were collected for further study. The tumor size and weight were lower in the Pt/Co + L group than in the other groups (Figure 5g,h; Figure S39, Supporting Information). To investigate the GPx‐like enzymatic activity, the relative level of GSH in tumor tissues was measured. As a result of the synergetic photothermal effect and enzymatic activities, compared with that in the control group, the level of GSH in the Pt/Co + L group was reduced by more than 72% (Figure 5i). Next, tumor tissue sections were prepared and stained with haematoxylin and eosin (H&E), Ki67 antibody, and TUNEL reagent to observe molecular changes. As shown in Figure 5j, the proliferation of tumor cells was inhibited, and there were many dead and apoptotic tumor cells in the Pt/Co + L group. These results demonstrate the good antitumor effects of the Pt/Co BNzyme. In addition, the major organs of the experimental mice were collected for pathological examination. Compared with the control, the Pt/Co BNzyme caused no obvious structural damage or changes in the major organs, supporting its biosafety (Figure S40, Supporting Information).

### 2.5 Pt/Co BNzyme Combined with αPD‐L1 Could Induce ICD to Improve the Immune Response and Relieve the Immunosuppressive State

**Figure 6:**
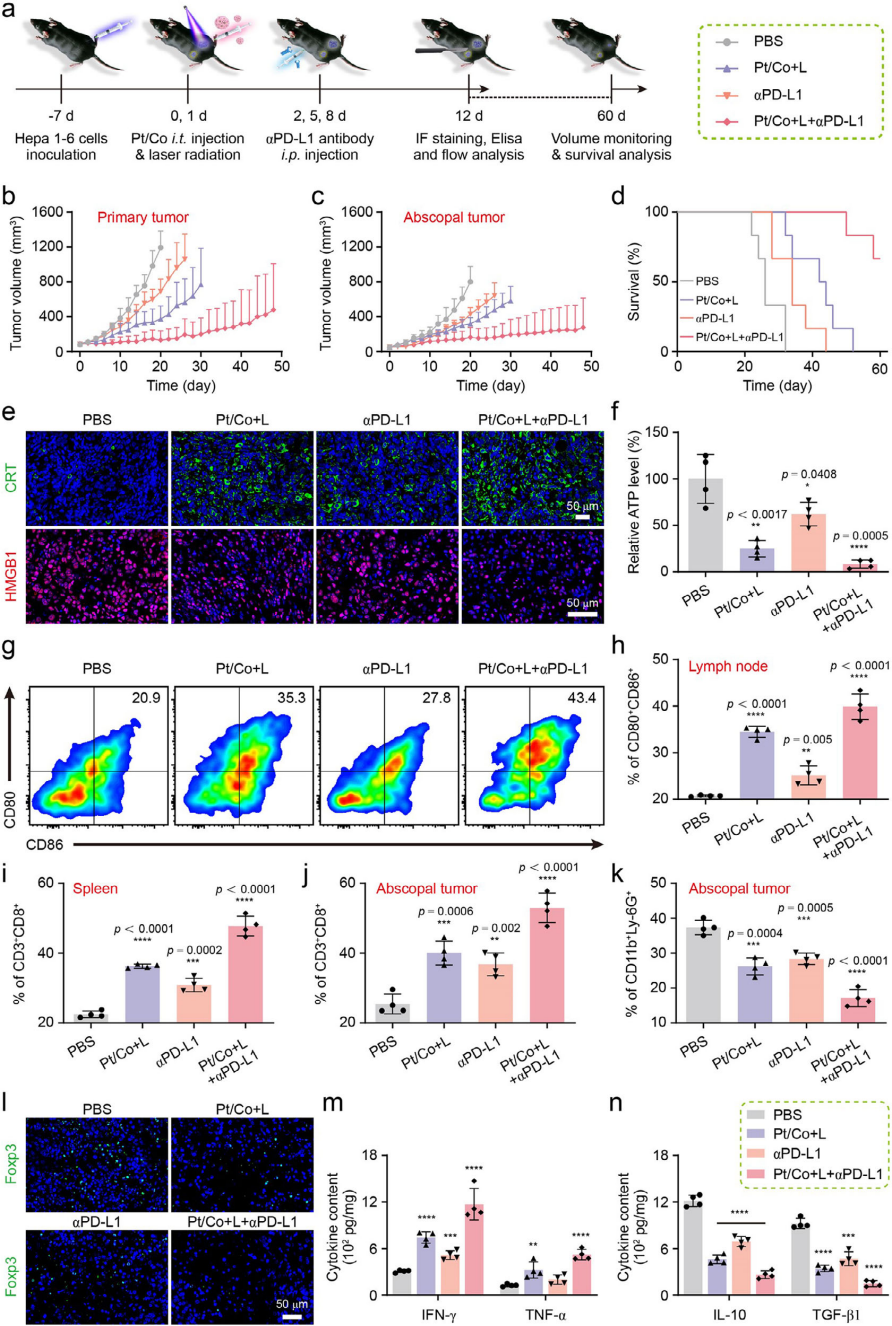
Pt/Co BNzyme combined with an ICI induced ICD to promote an immune response and relieve the immunosuppressive state in a bilateral tumor model. a) Flow chart of the combined therapy and analyses. Changes in the volume of primary tumors b) and abscopal tumors c) in mice subjected to various treatments. d) The survival rate of tumor‐bearing mice subjected to various treatments for 60 days. e) Immunofluorescence images of CRT and HMGB1 expression in primary tumors subjected to various treatments. Scale bar = 50 µm. f) Relative ATP content in primary tumors subjected to different treatments compared with that in the PBS group. g) CD80^+^CD86^+^ mature DCs in the DLNs of primary tumors detected by flow cytometry. h) Average maturation rates of DCs in the different treatment groups shown in Figure 6g. i) The percentage of CD3^+^CD8^+^ T cells in the spleen, as determined by flow cytometry. The rates of CTLs j) and MDSCs k) in abscopal tumors subjected to different treatments. l) Immunofluorescence images of Foxp3^+^ Tregs (green) in abscopal tumors. Scale bar = 50 µm. The content of IFN‐γ/TNF‐α m) and IL‐10/TGF‐β1 n) in abscopal tumors. The data are presented as the mean ± SD (n = 4; ^*^ *p* < 0.05, ^**^ *p* < 0.01, ^***^ *p* < 0.001, and ^****^ *p* < 0.0001).

To further study the immunological regulatory effects of the Pt/Co BNzyme, we constructed a bilateral tumor model in C57BL/6 mice. Different concentrations of Hepa1‐6 tumor cells were inoculated into both hips of each mouse: the tumor that developed on the side with more injected cells was called the primary tumor, and the other was referred to as the abscopal tumor. The mice were subsequently divided into four groups to receive different treatments: PBS, Pt/Co + L, αPD‐L1 and Pt/Co + L + αPD‐L1. Fifty microlitres of 1 mg mL^−1^ Pt/Co BNzyme was injected directly into the primary tumor, which was subsequently irradiated after 12 h (808 nm, 0.6 W cm^−2^, 10 min). One hundred micrograms of αPD‐L1 antibody were administered by intraperitoneal injection at 0, 3 and 6 d. Then, 4 mice were sacrificed for immune analysis at 10 d, and 6 mice were monitored for survival over 60 days (**Figure** **6**a). As shown in Figure 6b,c, and Figure S41 (Supporting Information), the primary and abscopal tumors grew quickly without treatment, and all six mice died at 32 d (Figure 6d). ICIs are among the most important tumor therapies, but the immunosuppressive tumor microenvironment limits their effectiveness.^[^ ^55^, ^56^ ^]^ The use of αPD‐L1 alone delayed tumor growth, but all of these mice died at 44 d. As a result of its synergetic effects, the Pt/Co BNzyme could directly kill the primary tumor, with an effect better than that of the ICI. In addition, the abscopal tumor was inhibited, which might be due to the activation of the immune response, and the longest survival time in the Pt/Co + L group reached 52 d. In combination with the ICI, the growth of tumors was effectively decreased in the Pt/Co + L + αPD‐L1 group (Figure S42, Supporting Information). Compared with those in the other groups, the primary and abscopal tumors in the combination group were significantly smaller at 20 d (*p* < 0.0001; Figure S43, Supporting Information). The median mortality rate was not reached by 60 d in the combined treatment group, indicating better survival. Moreover, no significant change in body weight was observed during treatment (Figure S44, Supporting Information).

To explore the mechanism underlying this phenomenon, after treatment, the primary tumors were collected for analysis. As shown in Figure 6e, more CRT (green) was expressed on the cell membrane in the Pt/Co + L + αPD‐L1 group. With greater extracellular release of HMGB1 (red), the intracellular HMBG1 content was reduced. Similarly, the ATP content in the tissue homogenate was also significantly reduced (*p* = 0.0005; Figure 6f). In addition, compared with αPD‐L1 alone, Pt/Co + L induced ICD more effectively. In this process, DAMPs are recognised by DCs, stimulating DC maturation. These DCs subsequently pass through the lymphatic vessels to the draining DLNs. Thus, we isolated DCs from DLNs and analysed them by flow cytometry (Figure 6g; Figure S45, Supporting Information). The proportion of mature DCs was more than 34.5% in the Pt/Co + L group, which was higher than that in the control group. Under combined treatment with αPD‐L1, the maturation rate increased to 39.9%, and the antigen presentation process was effectively initiated (Figure 6h). Subsequently, DAMPs are presented to T cells in the spleen by mature DCs. Consistent with the previous results, the percentage of CD3^+^CD8^+^ T cells (cytotoxic T lymphocytes, CTLs) in the Pt/Co+L+αPD‐L1 group was almost 11.5% greater than that in the Pt/Co + L group (Figure 6i; Figures S46 and S47, Supporting Information). These results indicate that the Pt/Co BNzyme could induce ICD in the primary tumor, promoting the activation of antigen‐presenting cells, activating antigen‐specific T cells, and enhancing the immune response in mice.

Next, the immune context of abscopal tumors was investigated. Owing to the activation of the immune system in mice, more CTLs were recruited to the abscopal tumors, as verified by flow cytometry (Figure 6j; Figures S48 and S49, Supporting Information). Immunofluorescence also revealed greater CD4^+^/CD8^+^ T‐cell infiltration in the Pt/Co+L+αPD‐L1 group, which plays an important role in eliminating tumors (Figure S50, Supporting Information). However, owing to the TME and immune escape, immunosuppressive cells in tumors inhibit and deplete immune cells. Myeloid‐derived suppressor cells (MDSCs) can be activated by the death of tumor cells and significantly inhibit the response of immune cells.^[^ ^57^, ^58^ ^]^ The combined use of the ICI could affect this inhibition and enhance the efficacy of immunotherapy. The flow cytometry results revealed that the percentage of CD11b^+^Ly6G^+^ MDSCs was reduced by nearly 20.2% in the Pt/Co+L+αPD‐L1 group (Figure 6k; Figures S51 and S52, Supporting Information). Forkhead box protein P3 (Foxp3) is a specific marker of regulatory T cells (Tregs) and is closely related to the local immune tolerance and immune escape of tumors.^[^ ^59^, ^60^ ^]^ Immunofluorescence staining also revealed that the number of Foxp3^+^ Tregs (green) was reduced in the abscopal tumors in the Pt/Co+L+αPD‐L1 group (Figure 6l). Immunomodulatory cytokines in the TME can recruit immunosuppressive cells and support tumor development; at the same time, these immunosuppressive cells further increase the levels of cytokines.^[^ ^61^ ^]^ IL‐10 and TGF‐β1 are primarily responsible for immunosuppression and dominate the TME in a variety of cancers.^[^ ^62^ ^]^ As shown in Figure 6n, the levels of these two cytokines was significantly decreased in the abscopal tumors in the Pt/Co + L + αPD‐L1 group (*p* < 0.0001). In addition, the expression of interferon‐gamma (IFN‐γ, an immune‐activating factor) and TNF‐α (a proinflammatory factor), which are vital for the immune response, was significantly increased in the combined treatment group (Figure 6m). In summary, treatment with the Pt/Co BNzyme combined with αPD‐L1 not only stimulates an immune response in primary tumors but also changes the TME and relieves the immunosuppressive state in abscopal tumors, prolonging the survival of mice. Owing to the robust immune‐mediated inhibition observed in distant tumors, we believe that the Pt/Co nanozyme can likewise achieve effective immunotherapy for deep‐seated orthotopic liver cancer. According to the literature,^[^ ^63^, ^64^ ^]^ Pt nanomaterials exhibit superior photothermal performance under 1064‐nm laser irradiation. Therefore, in our future work, we will employ longer excitation wavelengths to achieve more effective suppression of orthotopic liver cancer.

### 2.6 Transcriptome Sequencing Verified Immune Contexture Remodeling After Combined Treatment with the Pt/Co BNzyme and αPD‐L1

**Figure 7:**
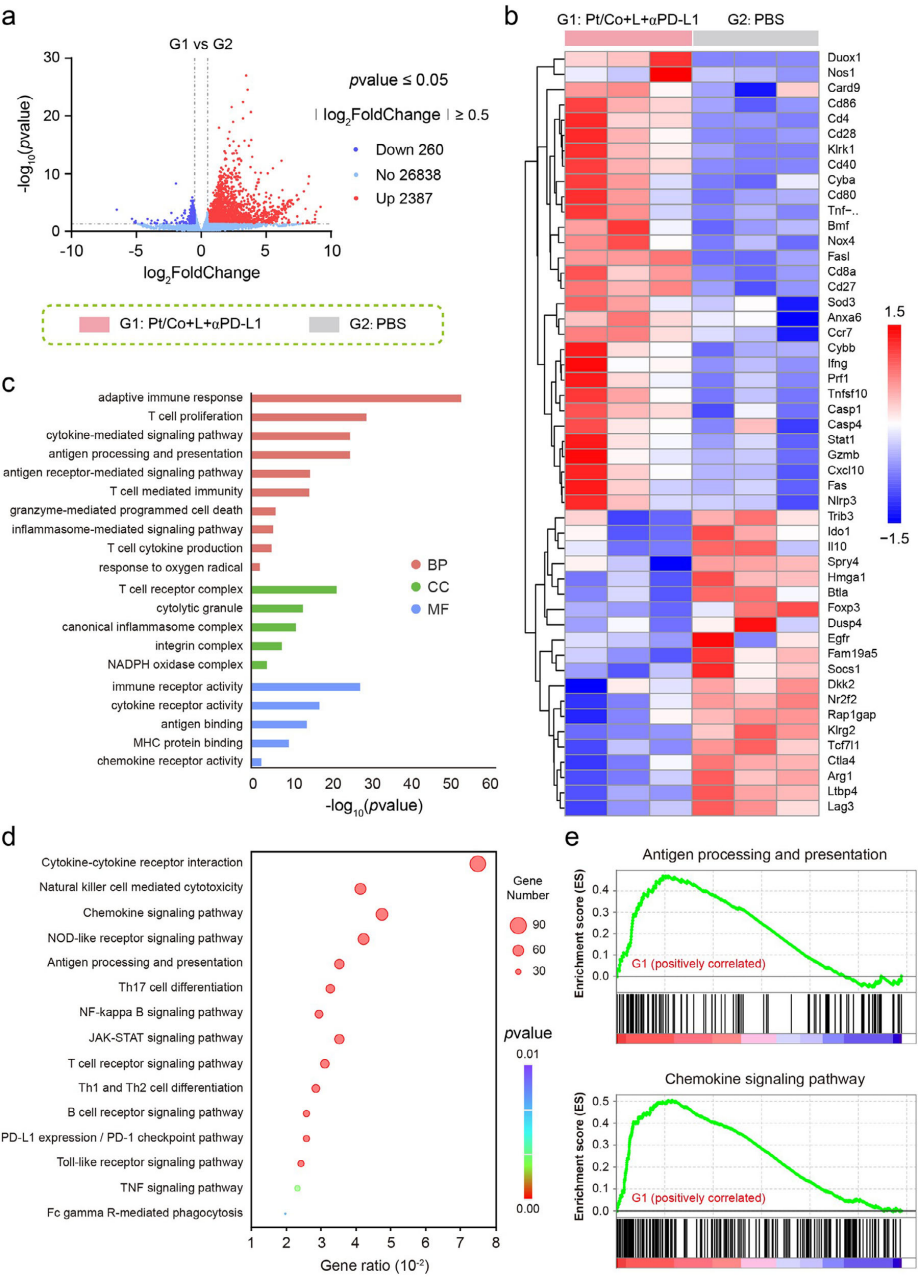
Transcriptome sequencing analysis of tumor tissues after treatment with Pt/Co‐BNzyme combined with an ICI. a) Volcano plot, b) heatmap analysis, c) GO enrichment analysis and d) KEGG enrichment analysis of DEGs between the PBS and Pt/Co + L + αPD‐L1 groups. e) GSEA enrichment analysis of positively related immunological pathways among the DEGs.

Phenotypic changes often suggest alterations in the gene expression of cells. Immune contexture remodeling was observed in the bilateral tumor model after combined treatment (Pt/Co + L + αPD‐L1). Therefore, we explored the molecular mechanism underlying this phenomenon by transcriptome sequencing. As shown in **Figure** **7**a, there were 260 downregulated genes and 2387 upregulated genes in the combined treatment group compared with the PBS group. The gene expression of the top differentially expressed genes (DEGs) was analysed by cluster analysis and is shown in a heatmap (Figure 7b). By categorical analysis, we found that a variety of immunological activities, such as immunogenic tumor cell death, dendritic cell maturation, T‐cell priming, and T‐cell‐mediated tumor cell elimination, were activated after combined treatment (Figure S53, Supporting Information). Gene Ontology (GO) analysis further revealed the biological process (BP), cellular component (CC) and molecular function (MF) terms of these DEGs. The key enriched BP terms included adaptive immune response, T‐cell proliferation, cytokine‐mediated signaling, antigen processing and presentation, and antigen receptor‐mediated signaling (Figure 7c). Kyoto Encyclopedia of Genes and Genomes (KEGG) enrichment analysis was performed to identify significantly altered pathways after combined treatment, including those involved in cytokine‒cytokine receptor interactions, natural killer cell‐mediated cytotoxicity, chemokine signaling, NOD‐like receptor signaling, and antigen processing and presentation (Figure 7d). Although these DEGs were located in corresponding pathways and biological functions, we could not determine whether the pathways were activated or inhibited by GO and KEGG analyses. Gene set enrichment analysis (GSEA) revealed the actual changes in all the pathways induced by the DEGs (Figure 7e). GSEA revealed that enrichment, antigen processing and presentation, chemokine signaling, PD‐1 checkpoint signaling, and T‐cell receptor signaling were upregulated in the combined treatment group (Figure S54, Supporting Information). The upregulation of these pathways could cause activation of the immune response, which is consistent with the results observed in the above‐mentioned animal experiments.

### 2.7 Pt/Co BNzyme Combined with αPD‐L1 Activated Immunological Memory to Inhibit Lung Metastasis

**Figure 8:**
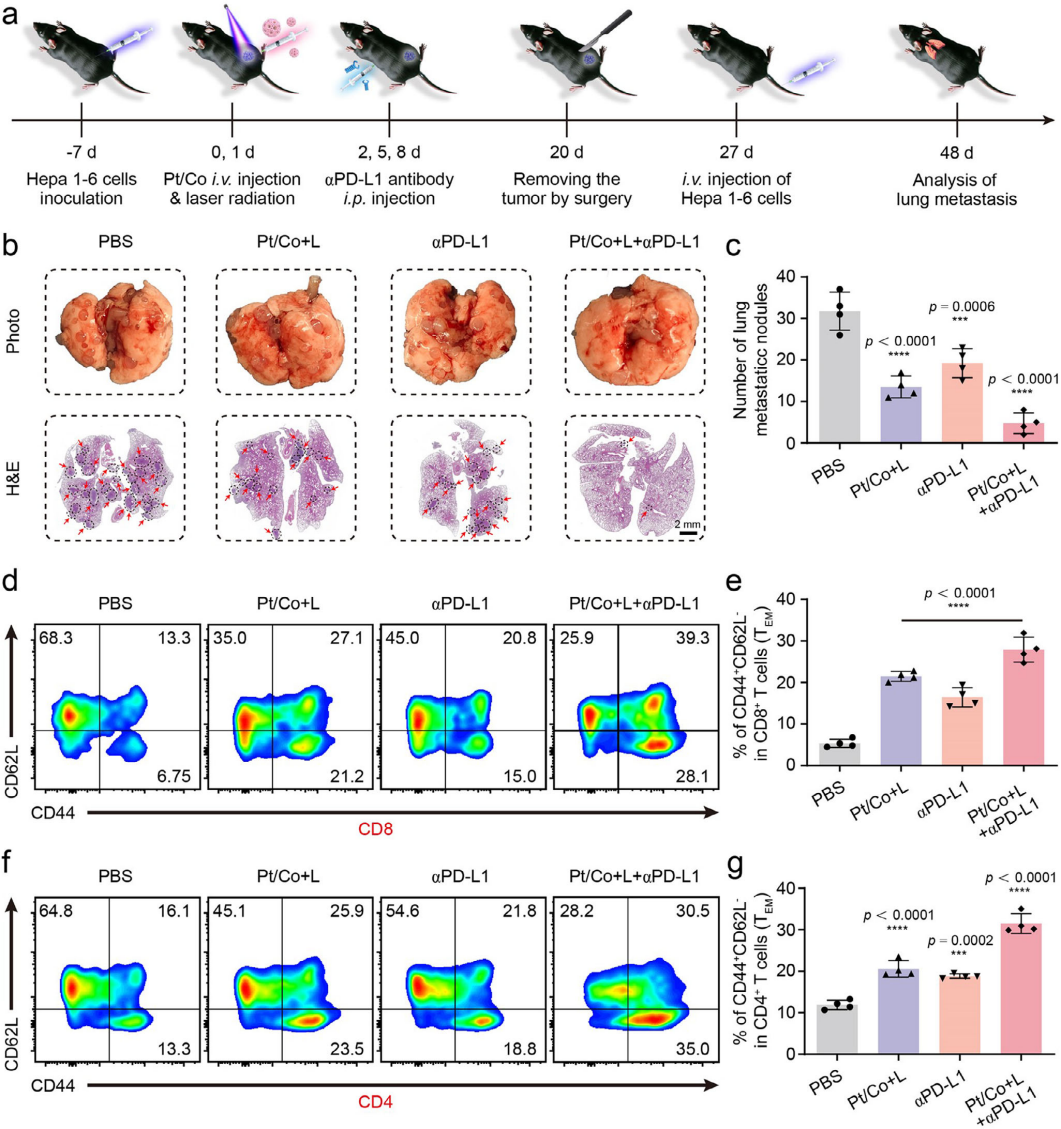
Pt/Co BNzyme combined with an ICI‐activated immunological memory to inhibit lung metastasis. a) Flow chart of the postoperative lung metastasis model. b) Photos and H&E staining of metastatic lesions in lungs after different treatments. Scale bar = 2 mm. c) Statistical analysis of the number of lung metastasis nodules in response to different treatments. d) Flow cytometry of memory T cells and e) the proportion of T_EM_ cells among CD8^+^ T cells. f) Flow cytometry of memory T cells and g) the proportion of T_EM_ cells among CD4^+^ T cells. The data are presented as the mean ± SD (n = 4; ^***^ *p* < 0.001 and ^****^ *p* < 0.0001).

Continuous immunological memory can help inhibit the recurrence and metastasis of tumors.^[^ ^65^ ^]^ C57BL/6 mice with subcutaneous tumors were first treated with the Pt/Co BNzyme, in combination with αPD‐L1 antibody or not (**Figure** **8**a). Then, the subcutaneous tumors were removed by surgery on day 20, and a lung metastasis model was constructed by injecting Hepa1‐6 cells through the tail vein. On day 48, the lungs of the mice were removed to observe the metastatic nodules. Numerous metastatic lesions appeared in the lungs of untreated mice, indicating that there was no chance of a cure (Figure 8b). Regardless of whether the Pt/Co BNzyme or ICI treatment alone was used, the inhibitory effects were barely satisfactory, and many metastatic lesions remained in the lungs. Only a few metastatic lesions were observed in the combination treatment group, indicating effective suppression of lung metastasis. The number of metastatic nodules was significantly lower than that in the other groups, suggesting that there was an opportunity for a cure (Figure 8c). These results might be attributed to the formation of immunological memory. Splenic cells from the mice were extracted and analysed by flow cytometry. Among the CD8^+^ T cells, the percentages of CD44^+^CD62L^−^ (effective memory T cells, T_EM_ cells) and CD44^+^CD62L^+^ (central memory T cells, T_CM_ cells) T cells were significantly increased in the Pt/Co + L + αPD‐L1 group (Figure 8d,e; Figures S55–S57, Supporting Information). Consistent with these results, the proportions of T_EM_ and T_CM_ cells among CD4^+^ T cells also significantly increased in response to the combined treatment (Figure 8f,g; Figures S58–S60, Supporting Information). These persistent immunological memory T cells could recognise and clear free tumor cells in the blood, which would reduce the probability of metastasis.

In this study, we successfully developed a Pt/Co mesoporous BNzyme with enzymatic activities and photothermal conversion capability. The synthesised Pt/Co BNzyme exhibited a uniform spherical morphology and long‐term stability, making it highly suitable for biomedical applications. Its POD‐like activity efficiently catalysed H_2_O_2_ to generate ·OH, while its GPx‐like activity depleted intracellular GSH, synergistically amplifying oxidative stress to induce tumor cell apoptosis. Furthermore, the photothermal performance under 808‐nm laser irradiation enabled a mild temperature increase to enhance the enzymatic activities and promote ICD. In vitro experiments confirmed the low cytotoxicity of the Pt/Co BNzyme. The combination of the enzymatic activities and mild photothermal effect achieved the greatest elimination of tumor cells via apoptosis. Importantly, the BNzyme triggered ICD, as indicated by CRT exposure, HMGB1/ATP release, and subsequent DC maturation, priming a systemic antitumor immune response. In vivo studies validated its biocompatibility and tumor‐targeting ability via the EPR effect. The Pt/Co BNzyme combined with NIR laser treatment significantly suppressed tumor growth and induced extensive apoptosis without harming normal tissues. When combined with an ICI (αPD‐L1), this therapy not only eradicated primary tumors by ICD but also inhibited abscopal tumor growth by remodeling the immunosuppressive TME, which included reducing IL‐10/TGF‐β1 expression, enhancing CTL infiltration, and inhibiting MDSCs and Tregs. Immunological memory (T_EM_/T_CM_ cells) was also activated by this combined therapy, which effectively prevented lung metastasis in a postoperative model. Transcriptomic analysis further corroborated the mechanisms of immune activation, highlighting upregulated pathways related to antigen presentation, T‐cell activation, and cytokine signaling. In summary, these findings support the potential of the Pt/Co BNzyme as a versatile nanoplatform for synergistic enzymatic and photothermal immunotherapy, providing a promising strategy for overcoming tumor resistance and immunosuppression.

## 3 Experimental Section

### Materials

All chemical reagents were obtained from commercial sources: K_2_PtCl_4_, CoCl_2_·6H_2_O, and L‐ascorbic acid (AA) were purchased from Frontier Scientific (USA). Poly(vinylpyrrolidone) (PVP, Mw = 40000)) was purchased from Sinopharm Chemical Reagent Co. (China). 3,3′,5,5′‐Tetramethylbenzidine (TMB), 2,7‐dichlorodihydrofluorescein diacetate (DCFH‐DA) and 5,5‐dithiobis(2‐nitrobenzoic acid) (DTNB) were obtained from Sigma‒Aldrich (USA). A CCK‐8 kit and calcein‐AM/propidium iodide (PI) were obtained from Dojindo Laboratories. Hyaluronidase, collagenase type IV and deoxyribonuclease were purchased from Vazyme (China). αPD‐L1 antibody was obtained from Proteintech (China).

### Synthesis of the Pt/Co BNzyme

In accordance with previously reported methods,^[^ ^22^ ^]^ mesoporous Pt/Co BNzyme was prepared as follows: 1 mL of ddH_2_O containing 0.3 mL of 20 mm CoCl_2_·6H_2_O, 0.7 mL of 20 mm K_2_PtCl_4_, and 0.01 g of PVP was stirred at room temperature for 10 min. Subsequently, 1 mL of 0.1 m AA was rapidly added, and the mixture was sonicated for 15 min at room temperature. The product was collected by centrifugation (10000 rpm, 20 min), washed three times with water via centrifugation, and then thermally treated in an oven at 200 °C for 12 h prior to XPS, XRD analyses and the following bioapplication.

### Characterisation of the Pt/Co BNzyme

Pt/Co BNzyme samples dispersed on carbon‐coated Cu grids were characterized using TEM/HAADF‐STEM (JEOL JEM‐2100F, 200 kV) coupled with EDS for elemental mapping. The hydrodynamic diameter and zeta potential were measured via DLS (Malvern Zetasizer Nano ZS) in ddH_2_O. XRD patterns were acquired on a Bruker D8 Advance (Cu‐Kα, λ = 1.54 Å, 10–80° 2θ range). Surface properties, including the BET surface area and BJH pore size distribution, were analysed by N2 adsorption/desorption (Micromeritics ASAP 2460). XPS spectra (Thermo Scientific ESCALAB 250Xi, Al‒Kα source) were calibrated to C 1s (284.8 eV).

### POD‐Like Enzymatic Activity Analysis

The POD‐like activity of the Pt/Co BNzyme was detected using TMB reagent. A mixture containing Pt/Co BNzyme (300 µg mL) and H_2_O_2_ (5 mm) in PBS (pH 6.0) was prepared. TMB solution (1 mm) was then added to initiate the reaction. The reaction proceeded at either room temperature or 43 °C, and the color changes associated with the colorimetric reaction were monitored at specific time points using a Vis‐NIR spectrophotometer.

### GPx‐Like Enzymatic Activity Assay

Pt/Co BNzyme (300 µg mL^−1^) and GSH (final concentration: 1.25 mm) were coincubated with H_2_O_2_ (final concentration: 5 mm) in PBS (pH 7.4) at either room temperature or 43 °C. After incubation for predetermined time intervals, DTNB was added to a final concentration of 0.05 mm. The reaction was allowed to proceed for 5 min at room temperature to ensure complete derivatisation of the remaining free thiol groups. The absorbance of the resulting TNB was then recorded at 412 nm using a UV–Vis spectrophotometer to quantify the residual GSH concentration.

### Photothermal Effect Assay

To evaluate the photothermal conversion efficiency of the Pt/Co BNzyme under NIR irradiation, aqueous samples were prepared by combining 1 mL of ddH_2_O with 1 mL of the Pt/Co BNzyme solution. The mixture was then subjected to 808‐nm laser irradiation at a specific power density (0.4, 0.6, or 0.8 W cm^−2^) for 10 min. The real‐time temperature change within each solution during irradiation was continuously monitored by a probe, and photos were taken using an infrared thermal imager (FLIR E60).

### Cell Culture

Mouse hepatocellular carcinoma cells (Hepa1‐6, RRID:CVCL_0327) and normal mouse liver cells (BNL CL.2, RRID:CVCL_4383) were obtained from the American Type Culture Collection (ATCC). It is confirmed that all cell lines were free from contamination. Both cell lines were cultured in DMEM supplemented with 10% fetal bovine serum (FBS) and 100 IU/mL penicillin–streptomycin. The cells were maintained at 37 °C in a humidified atmosphere containing 5% CO_2_.

### Cellular Uptake

Hepa1‐6 cells were seeded into confocal dishes at a density of 2 × 10^5^ cells per well. Following attachment, the culture medium was replaced with fresh DMEM containing FITC‐labeled Pt/Co BNzyme at a final concentration of 300 µg mL^−1^. The cells were subsequently incubated with the BNzyme suspension for designated time intervals (0, 4, 8, and 12 h) under standard culture conditions. The cells were fixed with 4% paraformaldehyde (PFA) for 15 min and then stained with DAPI for 10 min. Finally, the cellular internalisation of FITC‐labeled Pt/Co BNzyme was visualised using a confocal laser scanning microscope (Zeiss LSM 780).

### Cytotoxicity Assay

BNL CL.2 and Hepa1‐6 cells were seeded into 96‐well plates at a density of 1 × 10^4^ cells well^−1^ and incubated for 24 h. The cells were then treated with varying concentrations of Pt/Co BNzyme (0–200 µg mL^−1^) for an additional 24 h. After treatment, 100 µL of CCK‐8 working solution (10% CCK‐8 reagent in serum‐free medium) was added to each well. After the cells were incubated at 37 °C for 30 min in the dark, the absorbance was measured at 450 nm using a microplate reader to determine cell viability.

### Antitumor Activity In Vitro

Hepa1‐6 cells were seeded in 96‐well plates at 1 × 10^4^ cells well^−1^ and incubated for 24 h. The plates were divided into six experimental groups: PBS control, PBS+ H_2_O_2_+laser (PBS+H_2_O_2_+L), Pt/Co BNzyme (Pt/Co), Pt/Co BNzyme+laser (Pt/Co+L), Pt/Co BNzyme+H_2_O_2_ (Pt/Co+H_2_O_2_), and Pt/Co BNzyme+H_2_O_2_+laser (Pt/Co+H_2_O_2_+L). In accordance with the group assignments, Pt/Co BNzyme (200 µg mL^−1^) and/or H_2_O_2_ (100 µm) were added, followed by 12 h of incubation. Groups receiving laser treatment were then subjected to 808‐nm irradiation (0.6 W cm^−^^2^) for 10 min. After an additional 24 h of incubation, three assays were performed: A) Viability assessment: Cell viability was quantified using a CCK‐8 assay according to the above protocol. The absorbance was measured at 450 nm. B) Live/dead staining: Cells were stained with calcein‐AM/propidium iodide (PI) working solution (2 µm/4.5 µm in serum‐free DMEM) for 30 min at 37 °C. Live (green) and dead (red) cells were imaged using fluorescence microscopy. C) Apoptosis analysis: Cells were trypsinized, washed with cold PBS, and resuspended in 100 µL of binding buffer. Then, Annexin V‐FITC (5 µL) and PI (5 µL) were added. Following 15 min of incubation in the dark at RT, flow cytometry was immediately performed. Caspase‐3/7 activity was quantified using a caspase‐Glo 3/7 assay kit (Promega, USA) following the manufacturer's instructions. After the treatments described above and an additional 24 h of incubation, 100 µL of caspase‐Glo 3/7 reagent was added to each well containing 100 µL of culture medium in the 96‐well plates. The plates were gently shaken for 30 s and incubated at room temperature in the dark for 30–60 min. Luminescence was recorded with a microplate luminometer, and values were expressed as relative luminescence units (RLU).

### Verification of Enzymatic Function

Hepa1‐6 cells were seeded in 96‐well plates at a density of 1 ×10^4^ cells well^−1^. A) Intracellular ROS detection: After different treatments (PBS, PBS+H_2_O_2_+L, Pt/Co, Pt/Co+L, Pt/Co+H_2_O_2_, and Pt/Co+H_2_O_2_+L), the cells were washed with PBS and loaded with 10 µm DCFH‐DA in serum‐free medium (30 min, 37 °C). After the cells were washed with PBS, ROS‐generated fluorescence was detected using a fluorescence microscope. B) Intracellular GSH detection: Treated cells (groups: PBS, PBS+L, Pt/Co, and Pt/Co+L) were trypsinized and resuspended in 100 µL of PBS. The cells were lysed by repeated freezing and thawing with liquid nitrogen. After centrifugation (12000 × g, 15 min, 4 °C), the supernatants were reacted with 0.1 mm DTNB for 10 min. The absorbance at 412 nm was measured using a microplate reader, after which GSH standard were used for quantification.

### Immunogenic Cell Death Markers

To detect immunosuppressive cell death markers, CRT/HMGB1 immunofluorescence staining was performed by fixing cells with 4% PFA, permeabilizing them with 0.1% Triton X‐100, and then staining them with anti‐CRT (Abcam ab2907, 1:200) or anti‐HMGB1 (Abcam ab79823, 1:500) antibody overnight at 4 °C, followed by incubation with Alexa Fluor‐conjugated secondary antibodies. Image fluorescence was observed by confocal microscopy. For ATP/HMGB1 release analysis, cell supernatants were collected and evaluated using an ATP assay kit (Beyotime) via luminescence (560 nm) or an HMGB1 ELISA kit (Beyotime), after which the absorbance (450 nm) was measured. The controls included unstained cells for background subtraction.

### In Vitro DC Maturation Assay

BMDCs were isolated from 6‐week‐old male C57BL/6 mice using established protocols. Briefly, femurs and tibiae were flushed with cold PBS. Erythrocytes were lysed with lysis buffer, followed by two washes in PBS. The cells were cultured for 7 days in RPMI 1640 (10% FBS, 1% penicillin‒streptomycin) supplemented with 20 ng mL^−1^ recombinant murine GM‐CSF and 10 ng mL^−1^ IL‐4, and the medium was replenished on days 2 and 4. On day 7, loosely adherent immature DCs (iDCs) were harvested for subsequent experiments. To assess the immunological effects, a Transwell coculture system is established. Hepa1‐6 cells (4×10^4^) were seeded in the upper chambers, while iDCs (4×10^5^) were seeded in the lower chambers. Following 4 h of culture for adhesion, the Hepa1‐6 cells received the designated treatments. Treated tumor cells were then cocultured with iDCs to evaluate immune activation. Following 24 h of coculture, the DCs were harvested and stained with anti‐mouse CD11c‐APC, anti‐CD80‐PE, and anti‐CD86‐PE‐Cy7 antibodies. Flow cytometry analysis was performed by gating on CD11c^+^ populations. Simultaneously, the media from the lower chambers were centrifuged (300 × g, 10 min), and the supernatants were assayed for TNF‐α and IL‐12 levels using ELISA kits according to the manufacturer's protocol. The absorbance was measured at 450 nm.

### Antitumor Efficacy in a Subcutaneous Model

C57BL/6 mice were obtained from SLAC Animal (Shanghai, China) for in vivo experiments. All procedures were approved by the Animal Ethics Committee of Fujian Medical University (IACUC FJMU 2024‐Y‐1387). To establish tumors, 100 µL of a Hepa1‐6 cell suspension (5×10^6^ cells) was injected subcutaneously into the right flank. When the solid tumur volume reached 80–100 mm^3^, the mice were randomized into 4 treatment groups: 1) PBS, 2) PBS + NIR laser (PBS + L), 3) Pt/Co BNzyme (Pt/Co), and 4) Pt/Co BNzyme + NIR laser (Pt/Co+L). Pt/Co BNzyme (5 mg kg^−1^) was administered via tail vein injection in the corresponding treatment groups, and tumur volume was measured every two days using digital callipers. The tumur volume (V) was calculated as follows: V = (length × width^2^)/2. At the experimental endpoint, the tumurs were resected, photographed, and weighed. The tissue samples were immediately fixed in 4% paraformaldehyde for 24 h and then processed through a graded ethanol series and xylene before being embedded in paraffin. Sections (4 µm thick) were mounted on charged slides for sequential histological analysis by Ki‐67 immunohistochemistry and H&E and TUNEL staining. This multiplexed histological approach enabled comprehensive evaluation of therapeutic efficacy.

### Antitumor Efficacy in a Bilateral Tumor Model

To construct a bilateral tumor model, C57BL/6 mice received subcutaneous injections of Hepa 1–6 cells (100 µL of 5×10^6^ cells) in the right dorsal region to form primary tumors and 2×10^6^ cells in the left dorsal region to form abscopal tumors. When the primary tumors reached 80–100 mm^3^, the mice were divided into for treatment groups: 1) PBS, 2) Pt/Co+L, 3) αPD‐L1, and 4) Pt/Co+L+αPD‐L1. Pt/Co (50 µL, 1 mg mL^−1^) was injected *i.t*., and NIR (808 nm, 0.6 W cm^−2^, 10 min) irradiation was applied at 24 h after the injection. αPD‐L1 (10 mg kg^−1^) was injected *i.p*. Tumor volumes were measured every two days using callipers until the termination of the study on day 60. Excised tumors were embedded in paraffin for immunofluorescence analysis of HMGB1, CRT, CD4, CD8 and Foxp3 expression. Single‐cell suspensions from tumors were stained with the following fluorescent antibodies: anti‐CD3‐APC and anti‐CD8‐PE for T cells and anti‐CD11b‐APC and anti‐Ly‐6G/Ly‐6C‐PE for MDSCs. The spleens were isolated, and collected splenic cells were stained with anti‐CD3‐APC and anti‐CD8‐PE antibodies. Then, the lymph nodes (DLNs) were isolated to obtain the DCs, which were subsequently stained with anti‐CD11b‐APC, anti‐CD80‐PE and anti‐CD86‐PE‐Cy7 antibodies. These cells were then analysed by flow cytometry according to the abovementioned methods. The contents of ATP and cytokines (including IL‐10, TGF‐β1, TNF‐α, and IFN‐γ) inside tumor tissues were determined using ELISA kits according to the manufacturer's instructions.

### Transcriptome Sequencing

For transcriptome sequencing, total RNA was extracted from tumor tissues using TRIzol reagent, followed by quality assessment (RNA integrity number > 7.0). Libraries were prepared with an Illumina TruSeq Stranded mRNA Kit (poly‐A selection) and quantified by Qubit. Paired‐end sequencing (150 bp) was performed on an Illumina NovaSeq 6000 (minimum 40 m reads/sample). Raw data were processed via FastQC (quality control) and aligned to the reference genome (GRCh38) using HISAT2. Differentially expressed genes (DEGs) were identified by DESeq2 (|log_2_FC| > 1, *p*‐adjusted < 0.05), followed by functional enrichment analysis (GO/KEGG) with ClusterProfiler and GSEA for pathway activation.

### Efficacy Against Lung Metastasis In Vivo

To establish the experimental model, C57BL/6 mice received a subcutaneous injection of 100 µL of a Hepa1‐6 cell suspension (5×10^6^ cells mL^−1^) in the right flank to induce primary tumor formation. When the tumor volume reached 80–100 mm^3^, the mice were randomly allocated into four treatment groups: 1) PBS, 2) Pt/Co+L, 3) αPD‐L1, and 4) Pt/Co+L+αPD‐L1. Primary tumors were surgically resected on day 20 after implantation. To investigate the metastatic potential, 5×10^5^ Hepa1‐6 cells in 100 µL of PBS were subsequently administered via tail vein injection. On day 48, the mice were euthanized for the evaluation of metastasis, and the lungs were harvested and fixed in 4% paraformaldehyde for H&E staining. Simultaneously, splenocytes were isolated and stained with antibodies against CD3‐APC, CD8‐PE, CD44‐PE‐Cy7, and CD62L‐PerCP‐Cy5.5 and then analysed using a flow cytometer.

### Statistical Analysis

All statistical analyses were performed using GraphPad Prism 8.0. For comparisons between two groups, two‐tailed unpaired Student's t tests were applied. Multiple groups were compared by one‐way analysis of variance (ANOVA). The data were presented as the mean ± standard deviation (SD) of at least three biologically independent replicates. The following significance thresholds were applied: ^*^ *p* < 0.05, ^**^ *p* <0.01, ^***^ *p* <0.001, and ^****^ *p* <0.0001.

## Conflict of Interest

The authors declare no conflict of interest.

## Supporting information



Supporting Information

## Data Availability

The data that support the findings of this study are available from the corresponding author upon reasonable request.
