# Supplementary material for: Mild Photothermal Bimetallic Mesoporous Nanozyme Triggers Immunogenic Cell Death and Immune Contexture Remodeling for Precision Hepatocellular Carcinoma Treatment
Source: Adv Sci (Weinh). 2025 Sep 25;12(46):e12578. doi: 10.1002/advs.202512578 (PMC12697843; doi:10.1002/advs.202512578)
Supplement: Supplementary file 1 — Supporting Information [file ADVS-12-e12578-s001.docx]

**Supplementary Information**

**Mild Photothermal Bimetallic Mesoporous Nanozyme Triggers Immunogenic Cell Death and** **Immune Contexture Remodelling for Precision Hepatocellular Carcinoma Treatment**

Yanbing Huang^#^, Lei Ding^#^, Yanbing Cao^#^, Qiang Feng, Yanjuan Li, Jianmin Wang, Zhixiong Cai, Fuli Xin^*^, Peiyuan Wang^*^, Jingfeng Liu^*^

Y. Huang, Q. Feng, J. Wang, F. Li, J. Liu

Innovation Center for Cancer Research, Clinical Oncology School of Fujian Medical University, Fujian Cancer Hospital, Fuzhou 350014, P. R. China.

E-mail: [drjingfeng@126.com](mailto:drjingfeng@126.com), dr_xinfuli@163.com

F. Li, J. Liu

Department of Hepatobiliary and Pancreatic Tumor Surgery, Clinical Oncology School of Fujian Medical University, Fujian Cancer Hospital, Fuzhou 350014, P. R. China.

L. Ding, Y. Cao, Y. Li, Z. Cai, P. Wang

The United Innovation of Mengchao Hepatobiliary Technology Key Laboratory of Fujian Province, Mengchao Hepatobiliary Hospital of Fujian Medical University, Fuzhou 350007, P. R. China.

E-mail: [wangpeiyuan@fjirsm.ac.cn](mailto:wangpeiyuan@fjirsm.ac.cn)

P. Wang

State Key Laboratory of Structure Chemistry, Fujian Institute of Research on the Structure of Matter, Chinese Academy of Sciences, Fuzhou 350002, P. R. China.

J. Wang

Fujian Key Laboratory of Advanced Technology for Cancer Screening and Early Diagnosis，Fujian Cancer Hospital, Fuzhou 350014, China.

L. Ding

School of Rare Earths, University of Science and Technology of China, Hefei 230026, P.R. China.

#These authors contributed equally: Y. Huang, L. Ding, and Y. Cao.

**Keywords**: Hepatocellular Carcinoma; Bimetallic Mesoporous Nanozyme; Photothermal Effect; Immunogenic Cell Death; Immune Contexture Remodeling


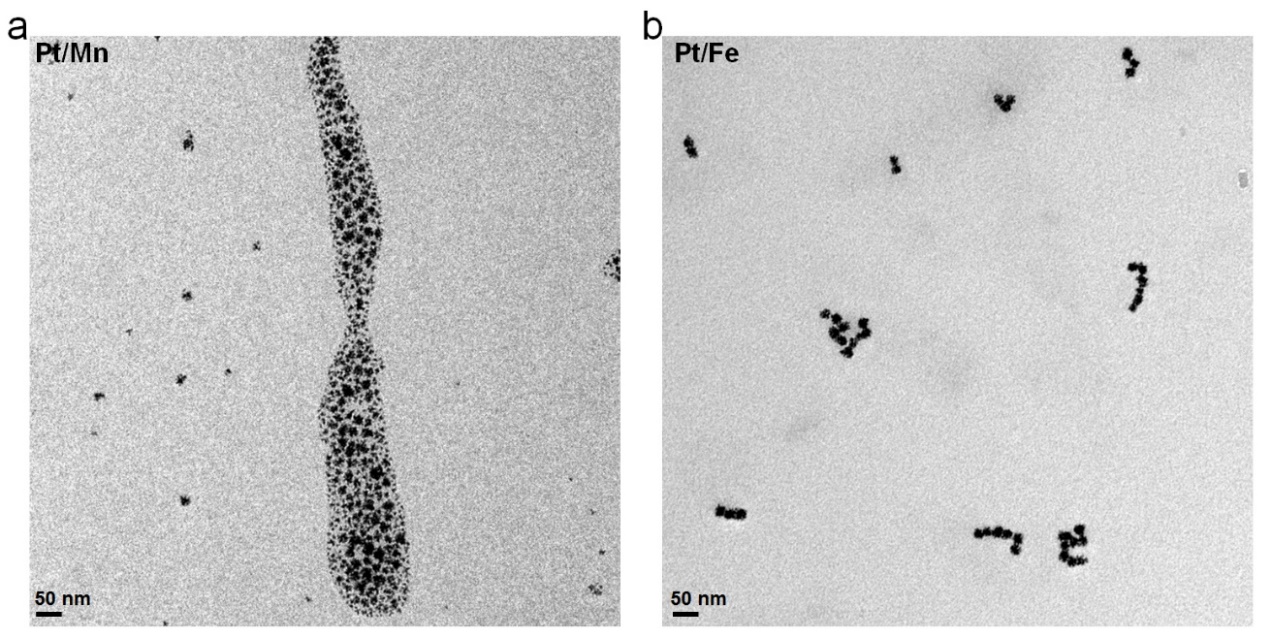


**Figure S1.** TEM images of Pt/Mn (a) and Pt/Fe (b).


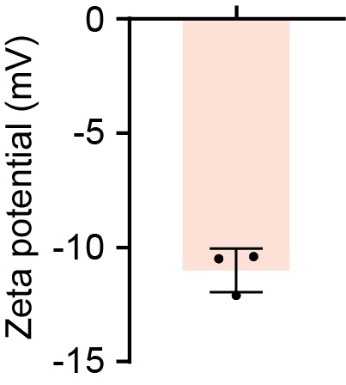


**Figure S2**. Surface zeta potential of Pt/Co BNzyme. The data are presented as mean ± SD (n = 4).


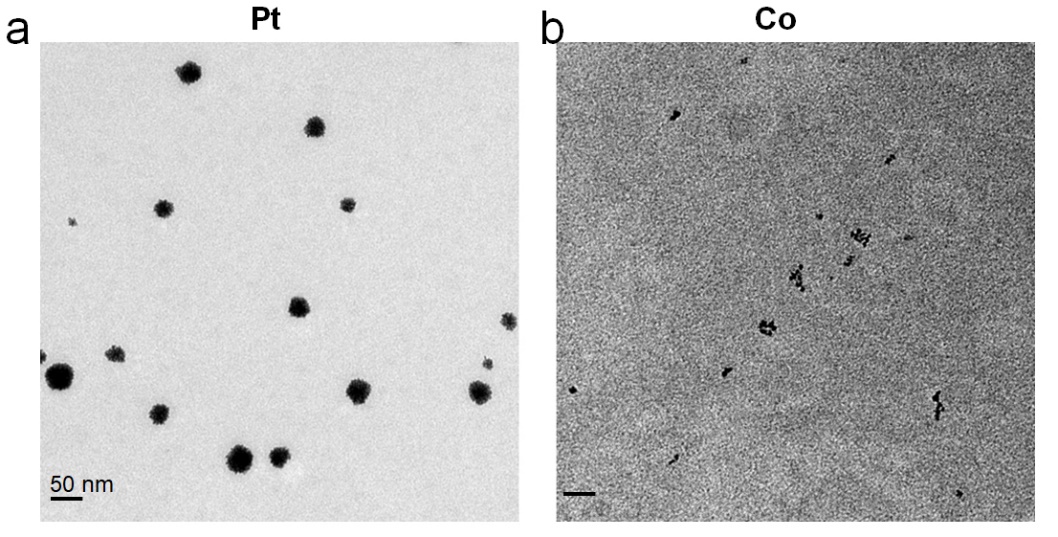


**Figure S3**. TEM images of (a) Pt and (b) when single Pt/Co precursor was used.





**Figure S4**. HRTEM of two Pt/Co nanoparticles.


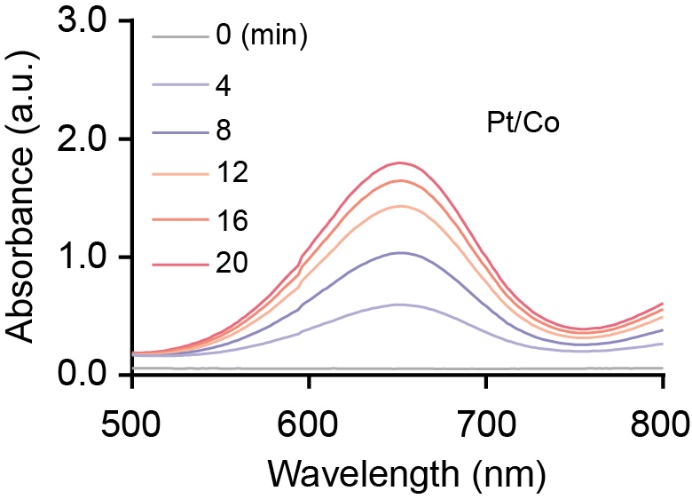


**Figure S5.** Time-dependent absorption spectra of TMB + H_2_O_2_ solution dealt with Pt/Co.

**
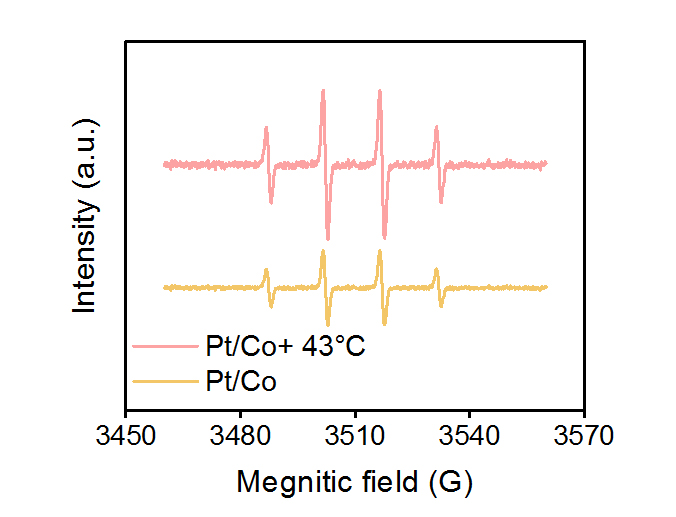
**

**Figure S6.** EPR spectra of Pt/Co and Pt/Co+43°C.

**
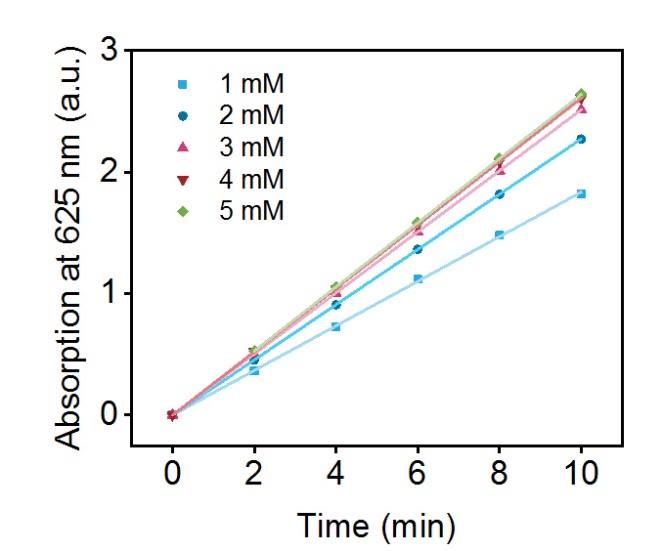
**

**Figure S7.** The absorbance values at 652 nm of Pt/Co+H_2_O_2_+TMB+43℃ system with fixed H_2_O_2_ concentration (5 mM) and varied TMB concentration (1-5 mM) for different minutes.


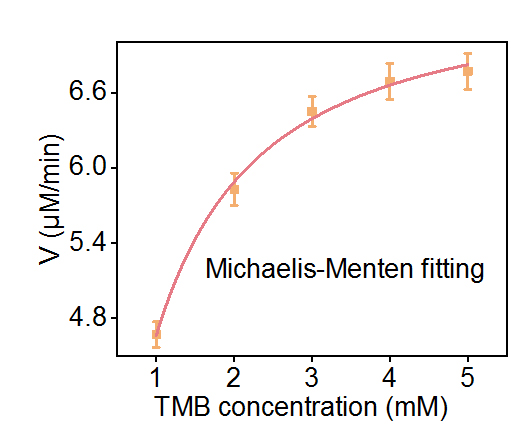


**Figure S8.** Michaelis-Menten curve for Pt/Co with TMB as substrate.


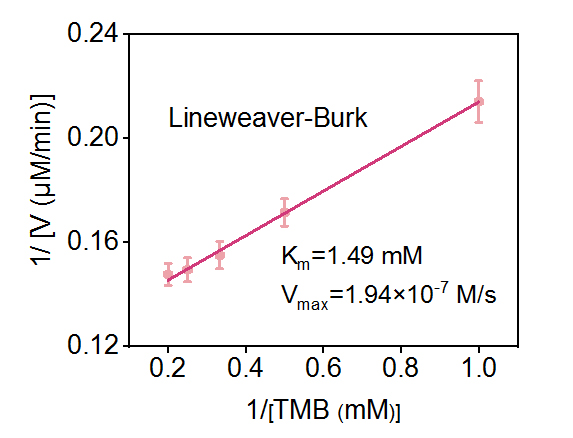


**Figure S9.** Lineweaver-Burk plot for Pt/Co with TMB.


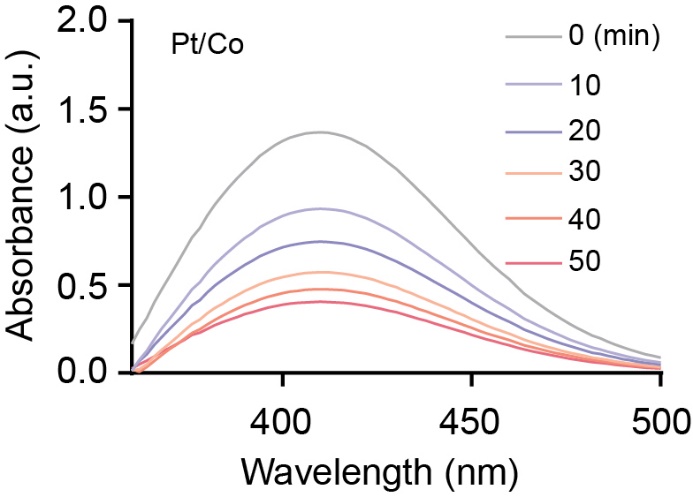


**Figure S10**. Time-dependent absorption spectra of GSH consumption dealt with Pt/Co detected by DTNB reagent.


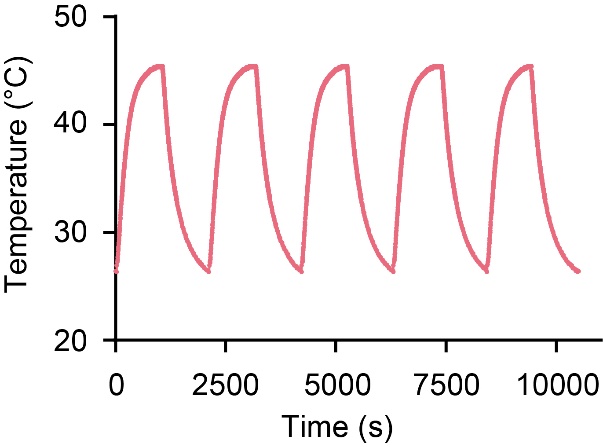


**Figure S11.** The heating profile of Pt/Co (200 μg/mL) over four cycles of laser on/off.


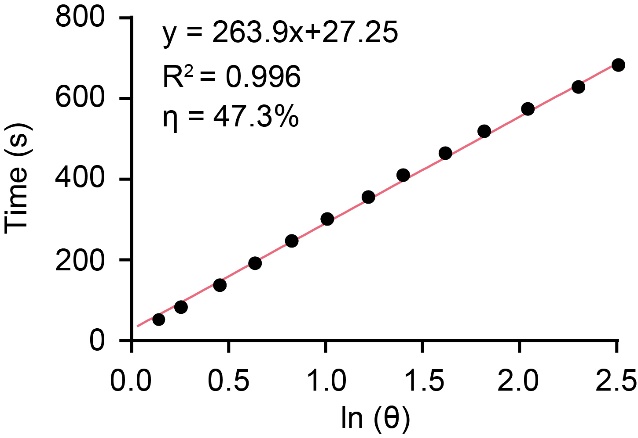


**Figure S12.** Linear relationship between the cooling time and -ln(θ).


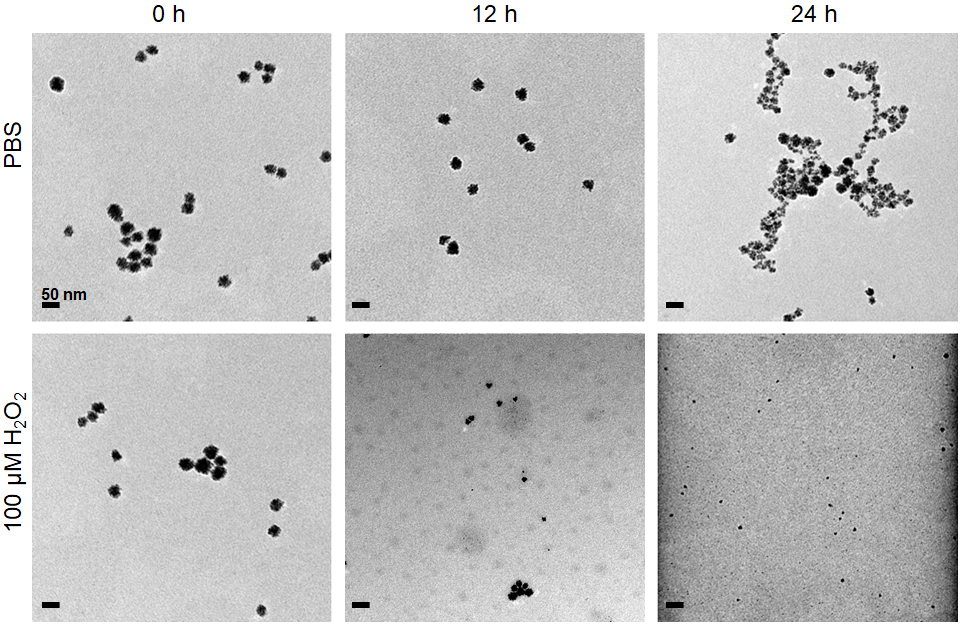


**Figure S13.** TEM images of Pt/Co nanoenzyme in the presence of PBS and H_2_O_2_ buffer for various period.


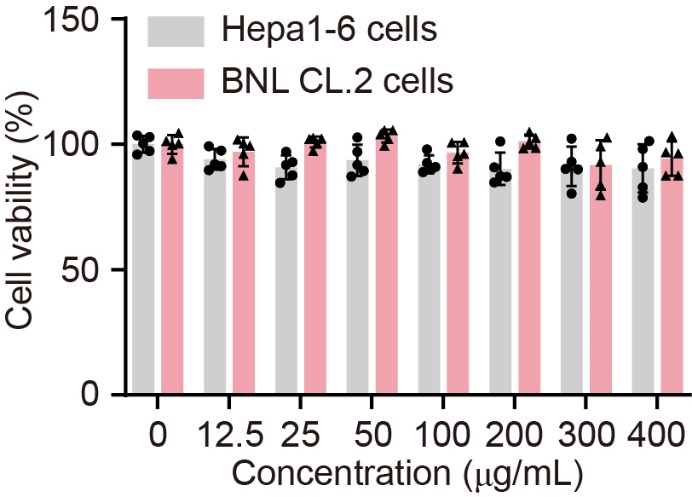


**Figure S14**. The cell viability of Hepa1-6 cells and BNL CL.2 cells incubated with Pt/Co BNzyme. The data are presented as mean ± SD (n = 5).


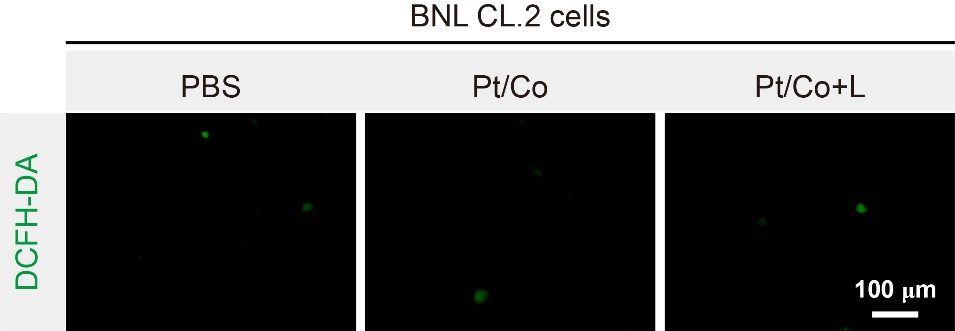


**Figure S15.** ROS generation in BNL CL.2 after treated with PBS, Pt/Co and Pt/Co+L.


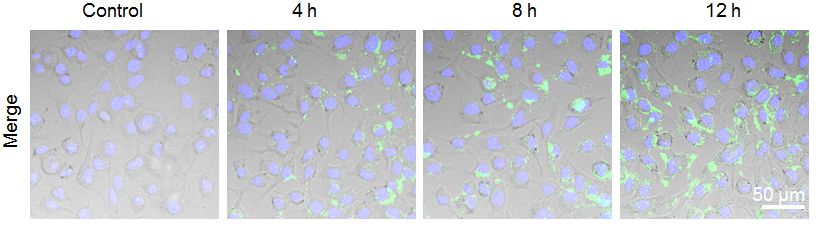


**Figure S16**. Bright field and fluorescence merged images of Pt/Co BNzyme incubated with Hepa1-6 cells for different durations.


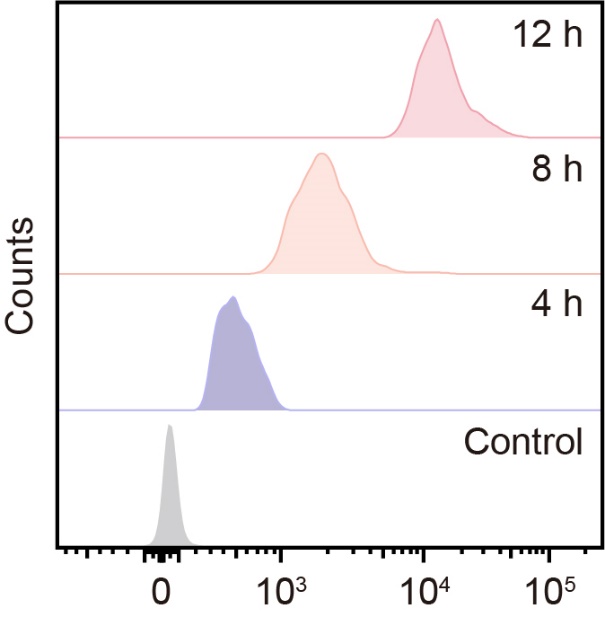


**Figure S17**. The uptake of Pt/Co BNzyme in Hepa1-6 cells detected by flow cytometry.


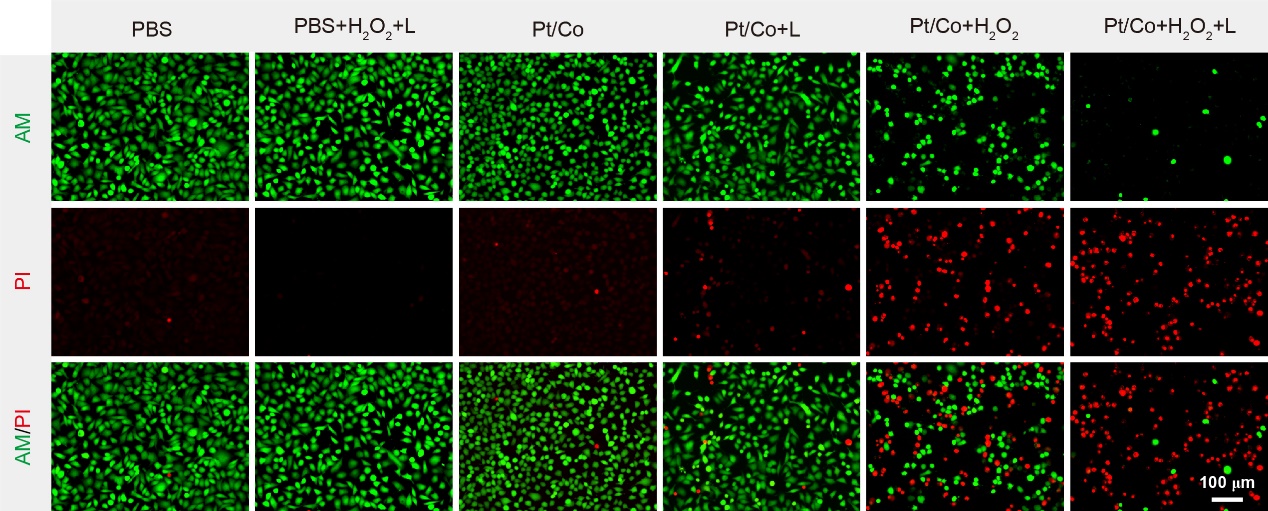


**Figure S18.** The live/dead state of Hepa1-6 cells dealt with different treatments and detected by AM/PI reagent. Scale bar = 100 µm.


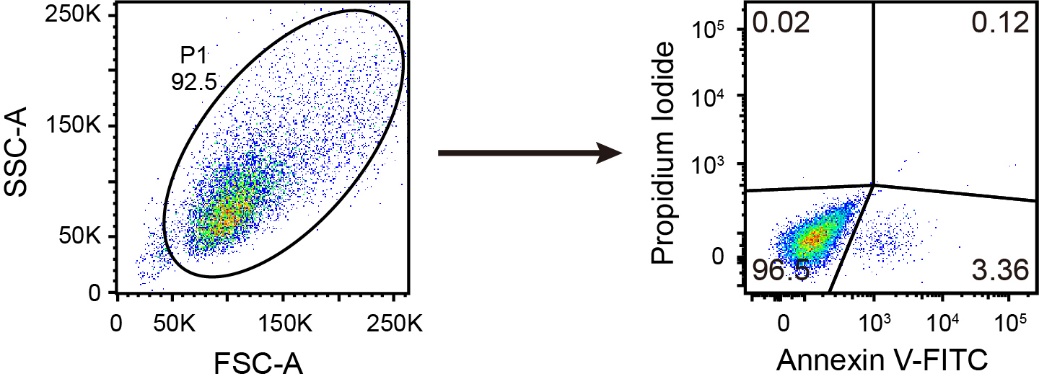


**Figure S19.** Gating strategy for analysis of apoptosis cells by Annexin-V/PI reagent.


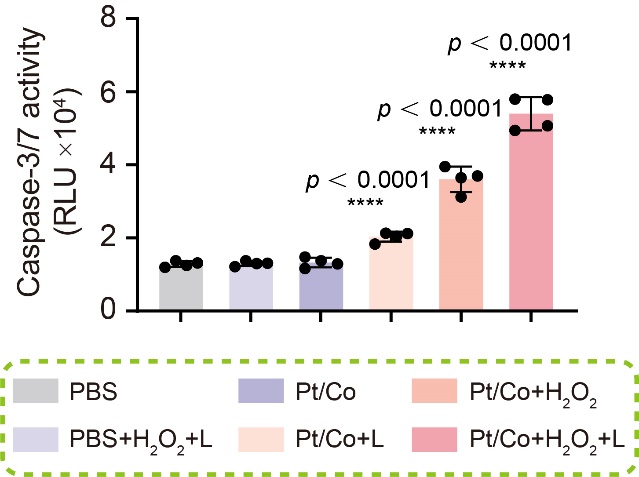


**Figure S20.** Caspase-3/7 levels in Hepa1-6 cells after different treatments.


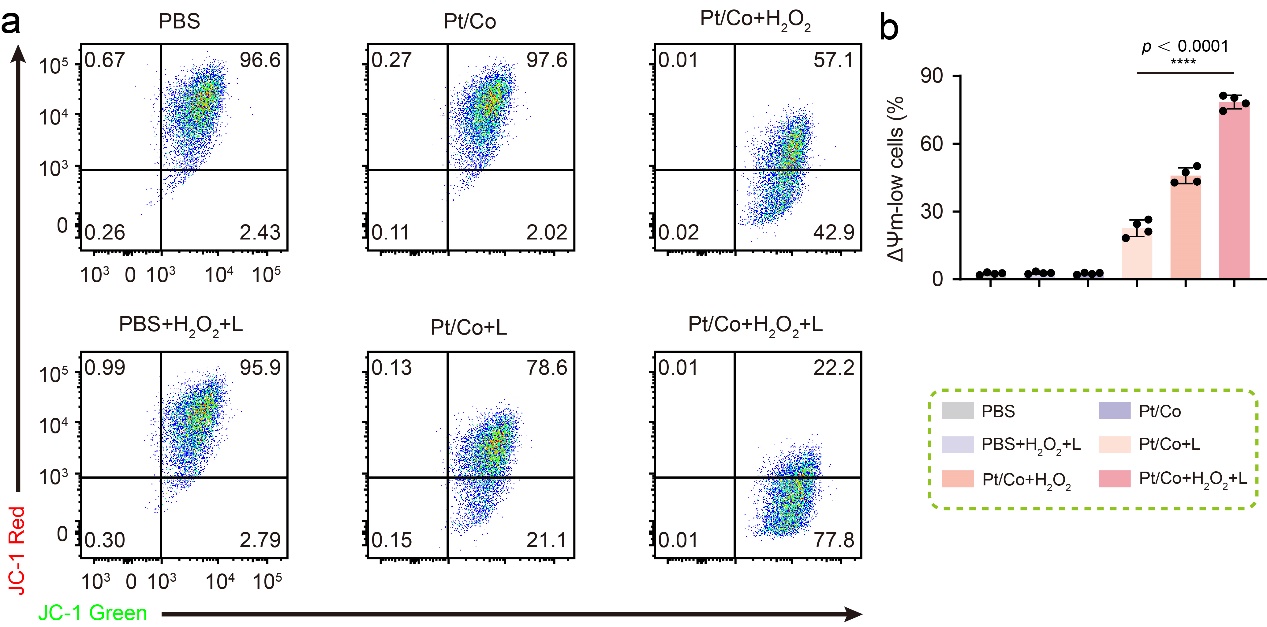


**Figure S21.** (a) Flow cytomter analysis of red and green fluorescence intensities from JC-1 stained Hepa1-6 cells after eight different treatments. (b) The mitochondrial membrane potential (ΔΨm) decreased percentage after various treatments.


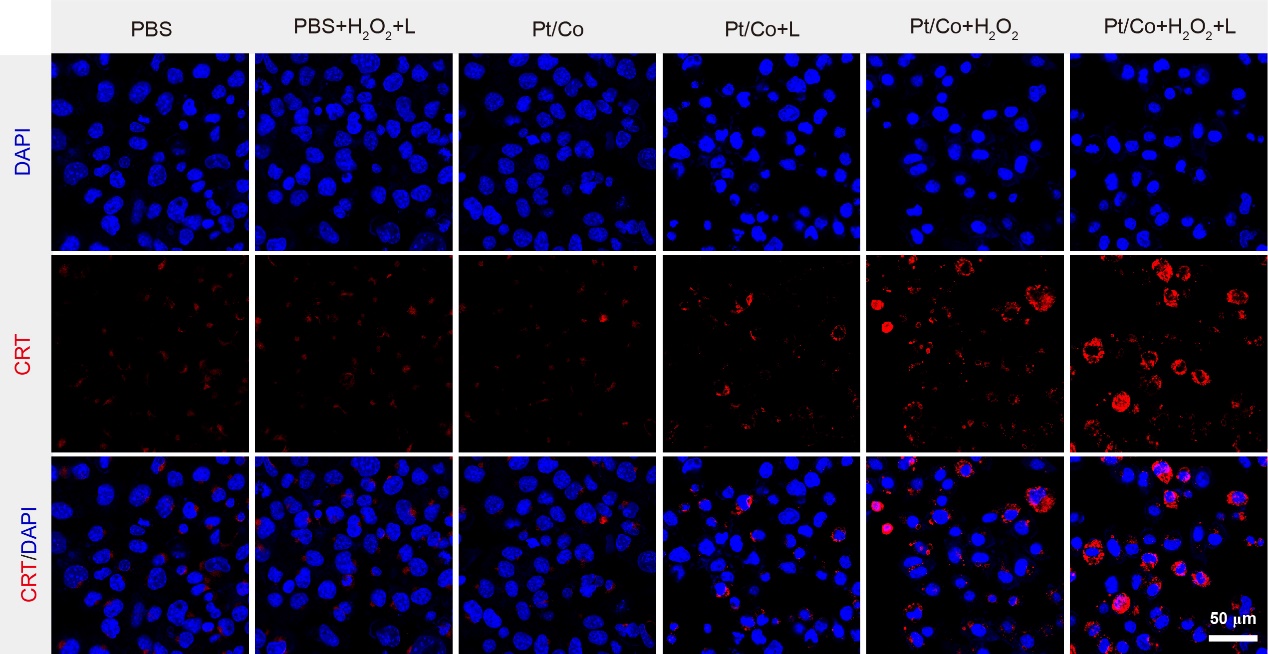


**Figure S22.** Immunofluorescence images of CRT expression in Hepa1-6 cells dealt with different treatments. Scale bar = 50 µm.


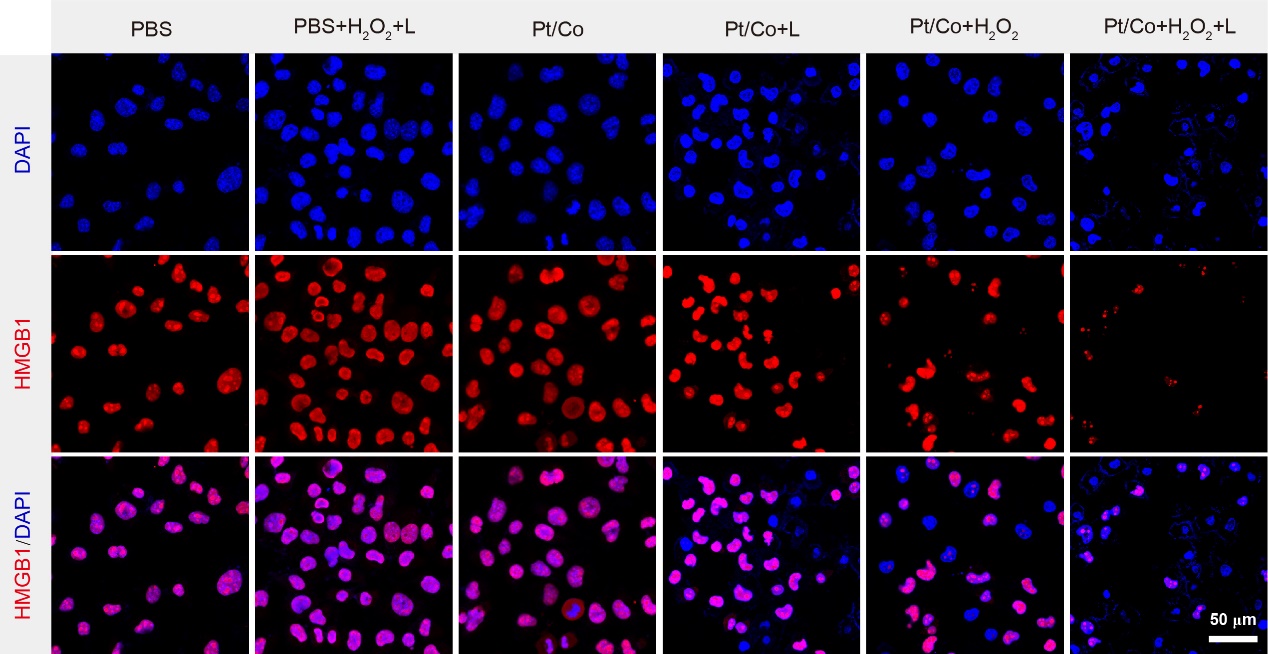


**Figure S23.** Immunofluorescence images of HMGB1 expression in Hepa1-6 cells dealt with different treatments. Scale bar = 50 µm.


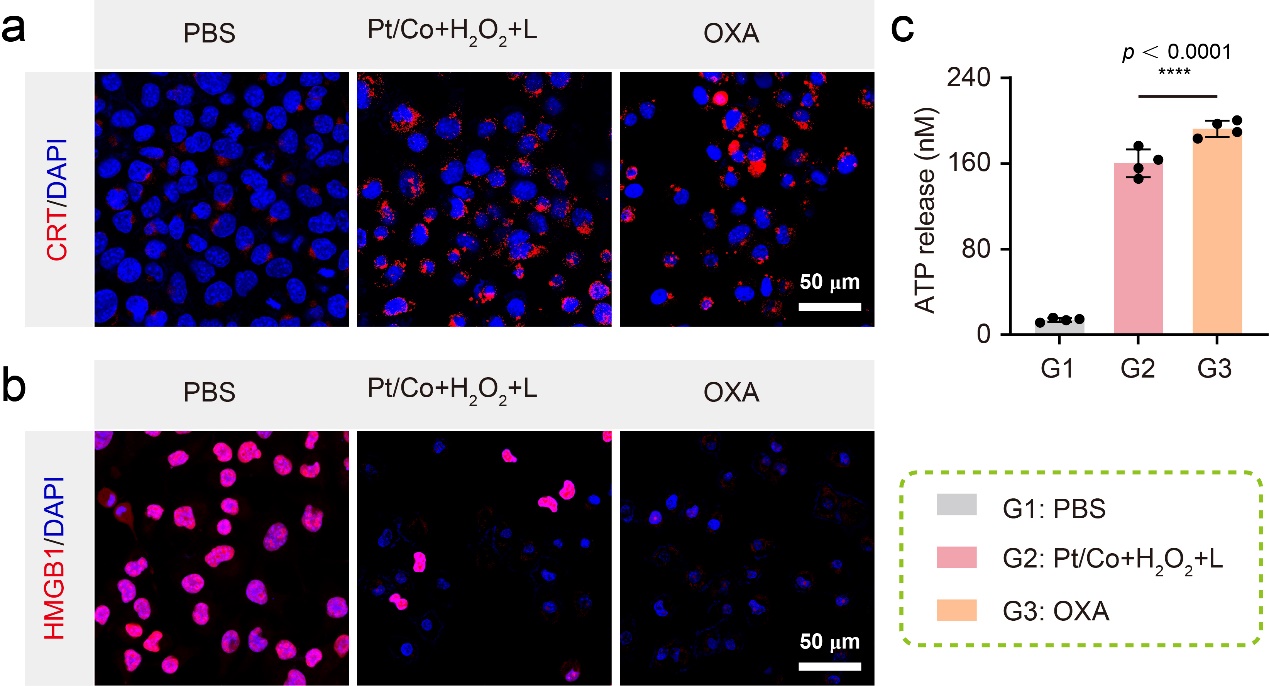


**Figure S24.** CLSM images of (a) HMGB1 and (b) CRT levels in Hepa1-6 cells after treated with PBS, Pt/Co+H_2_O_2_+L and oxaliplatin. (c) ATP released into the supernatant of Hepa1-6 cells dealt with different treatments.


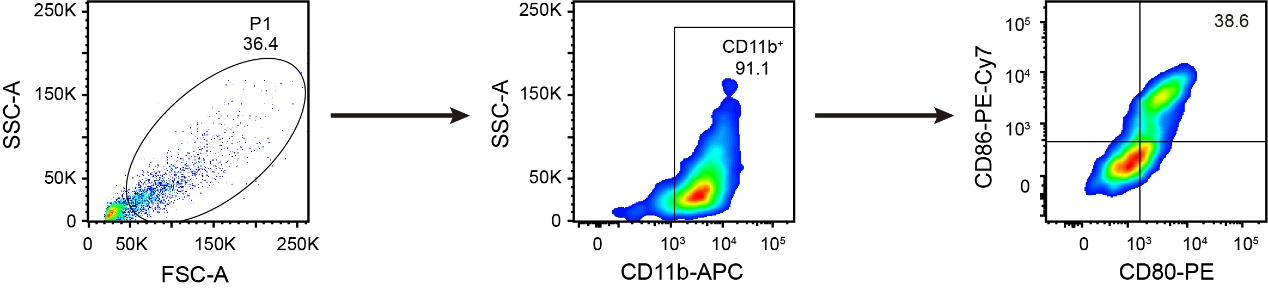


**Figure S25.** Gating strategy for analysis of mature DCs by CD11b, CD80 and CD86 antibody.


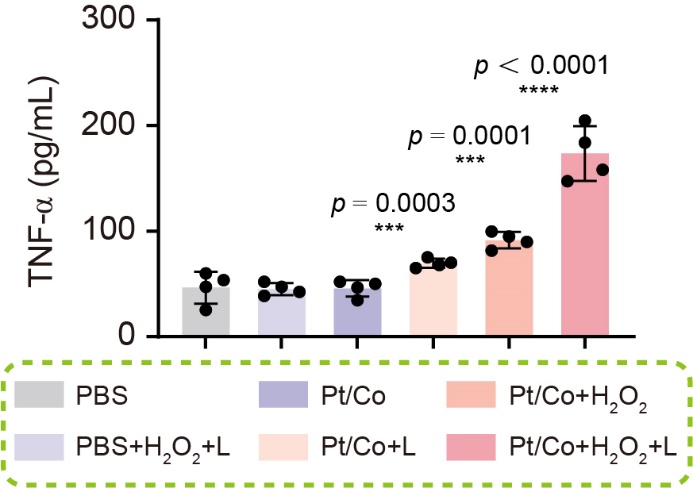


**Figure S26**. The content of secreted TNF-α cytokine in the supernatant related to Figure 4f tested by ELISA. The data are presented as mean ± SD (n = 4; ****p* < 0.001, *****p* < 0.0001).


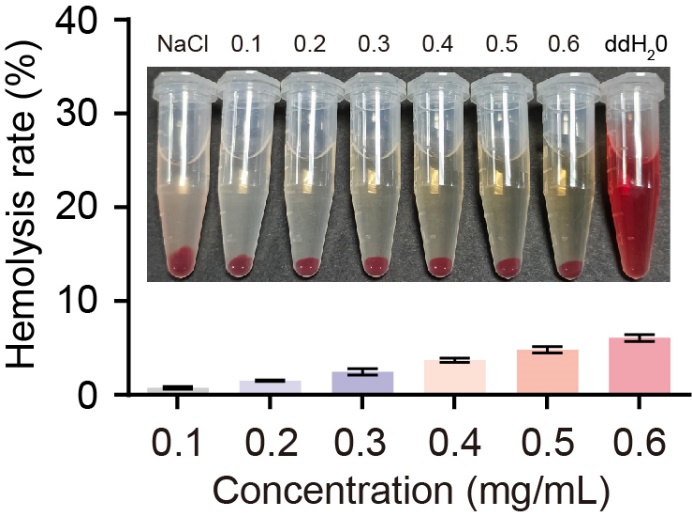


**Figure S27.** Hemolysis assay of Pt/Co BNzyme by incubating with mouse red blood cells at different concentration. The data are presented as mean ± SD (n = 4).


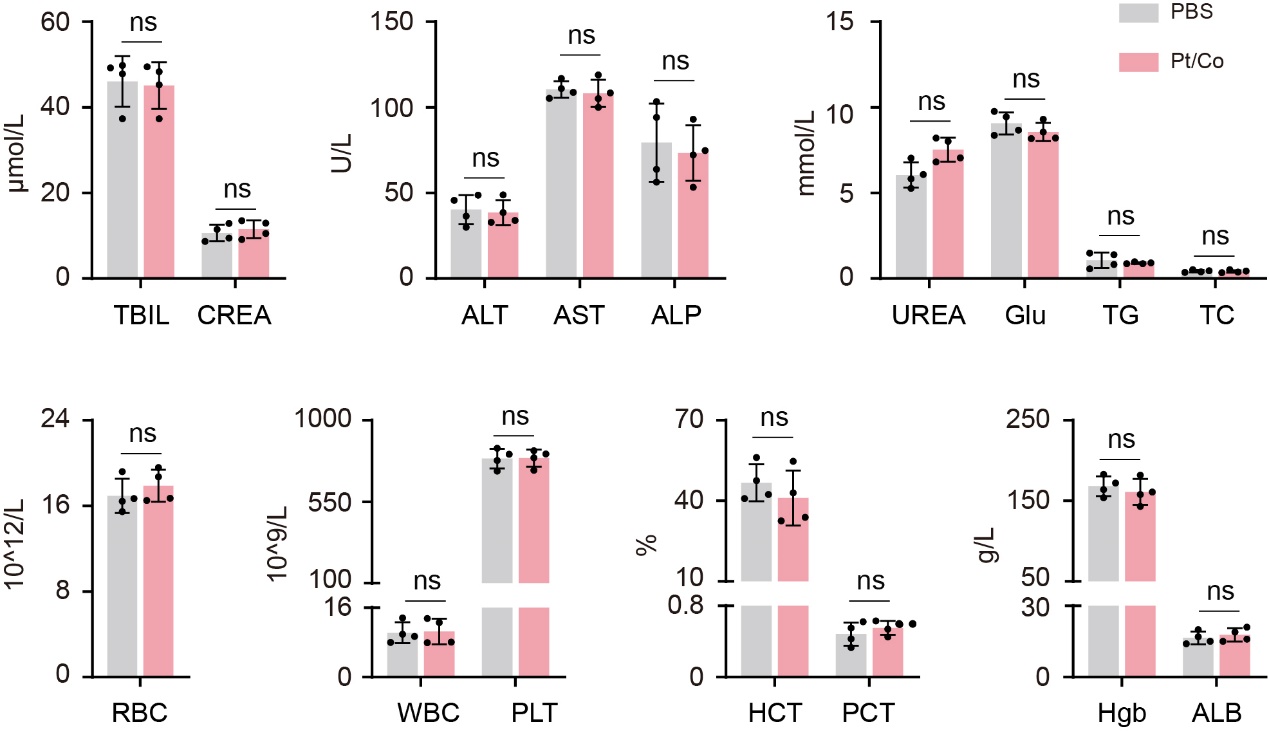


**Figure S28.** Blood cell and biochemical analysis of mice injected with PBS or Pt/Co BNzyme. The data are presented as mean ± SD (n = 4; ns = not significant).

**
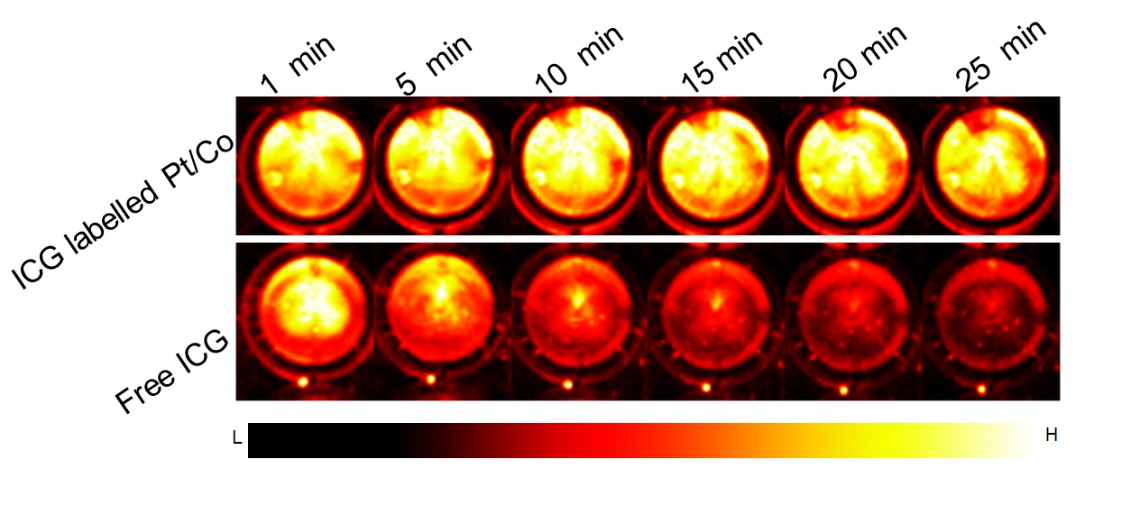
**

**Figure S29.** NIR II florescent pictures of ICG labelled Pt/Co and ICG after continuous laser irradiation for 25 min.


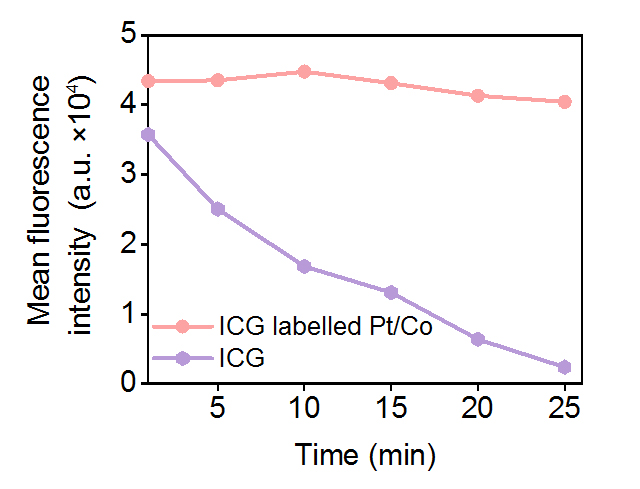


**Figure S30.** Quantitative signal intensity of ICG labelled Pt/Co and ICG after continuous laser irradiation for 25 min.


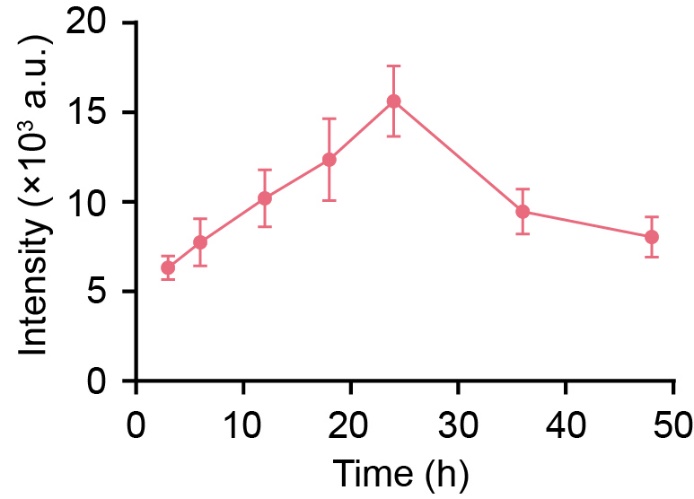


**Figure S31.** The change of fluorescence intensity in tumor site corresponding to Figure 5a. The data are presented as mean ± SD (n = 3).


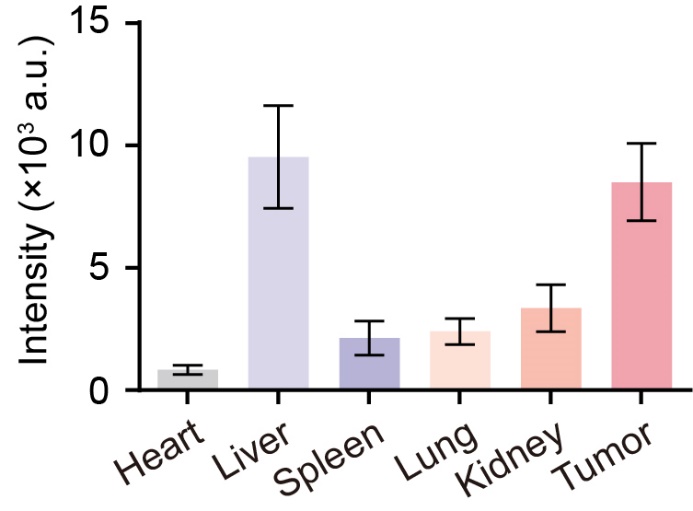


**Figure S32.** The intensity of tumor and major organs at 48 h corresponding to Figure 5b. The data are presented as mean ± SD (n = 3).


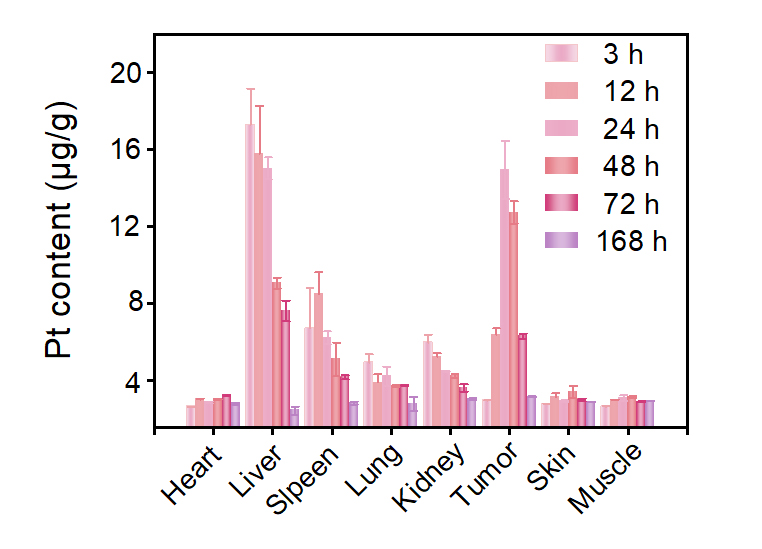


**Figure S33.** Pt contents in major organs and tumor after tail-vein injection of Pt/Co for various hours.


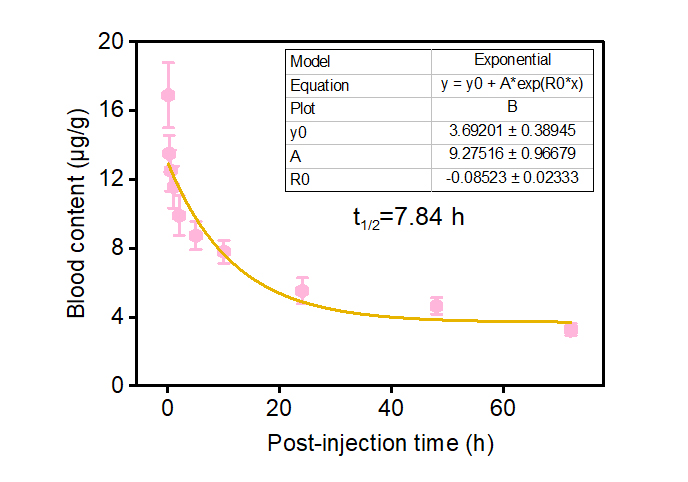


**Figure S34.** Blood circulation half-life of Pt/Co.


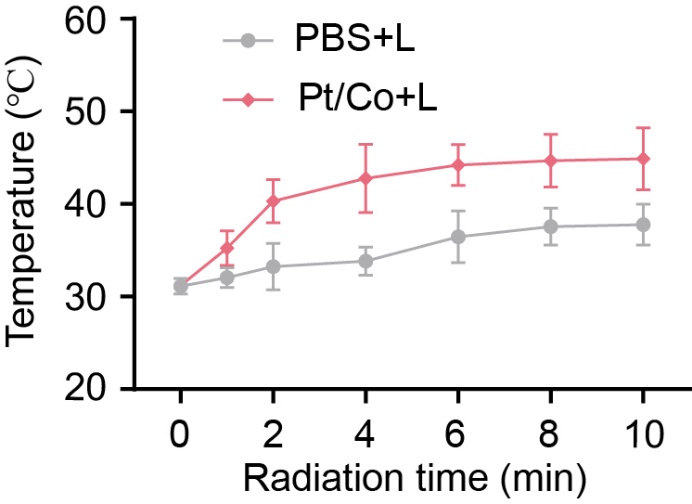


**Figure S35.** The temperature change of Hepa1-6 tumor irritated by NIR laser corresponding to Figure 5d. The data are presented as mean ± SD (n = 3).


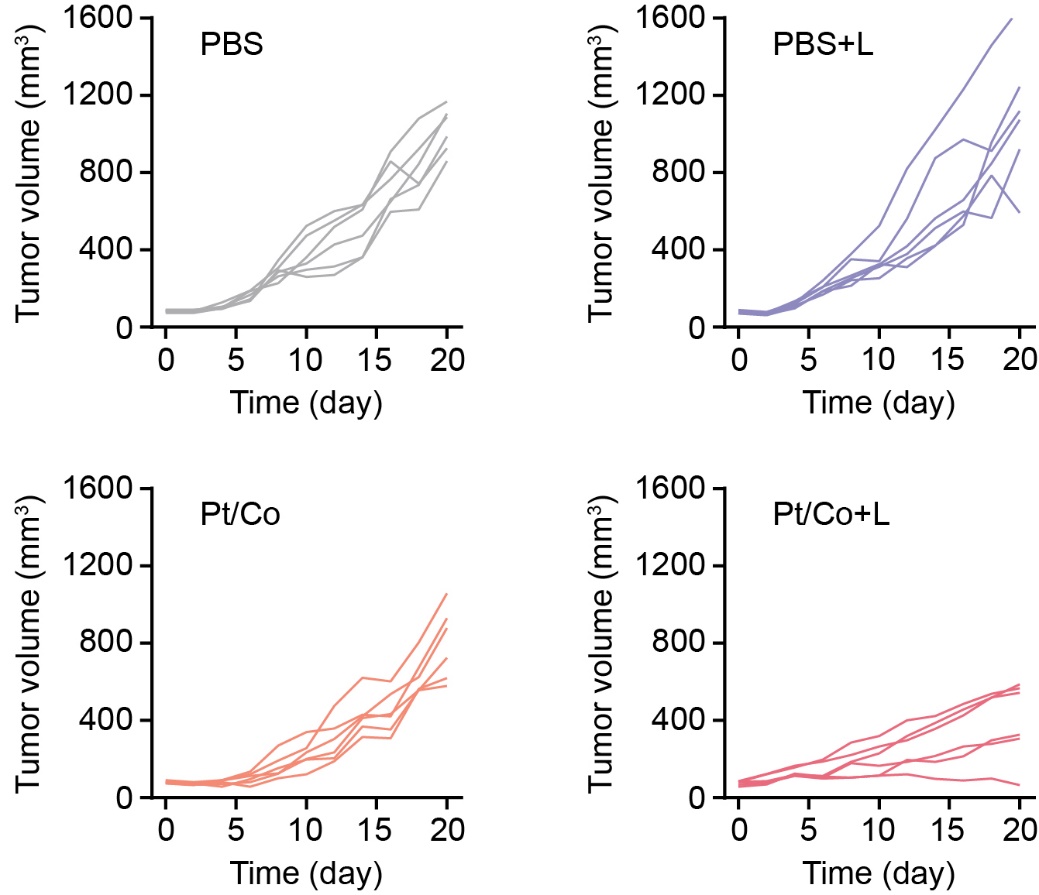


**Figure S36.** Tumor volume changes of each mouse with different treatments corresponding to Figure 5e.


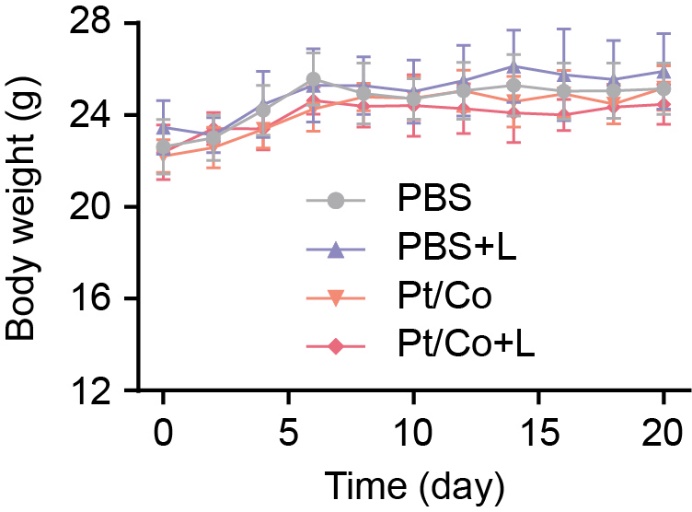


**Figure S37.** The body weight change of mice dealt with different treatments. The data are presented as mean ± SD (n = 6).


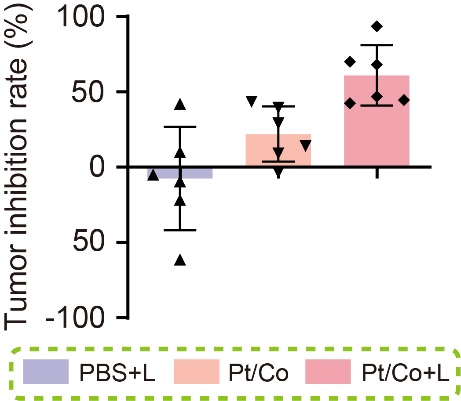


**Figure S38.** Tumor inhibition rates from subcutaneous Hepa1-6 tumor bearing mice.


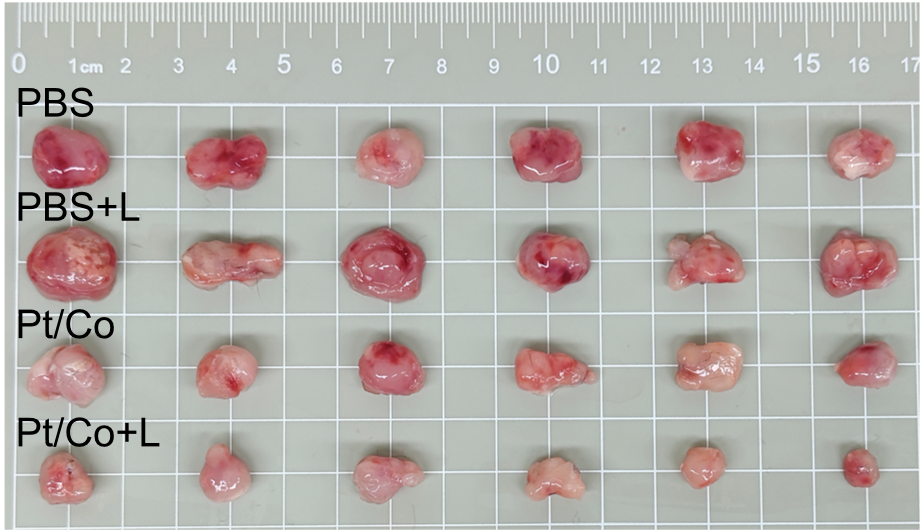


**Figure S39.** *Ex vivo* images of tumors dealt with different treatments at 20^th^ day.


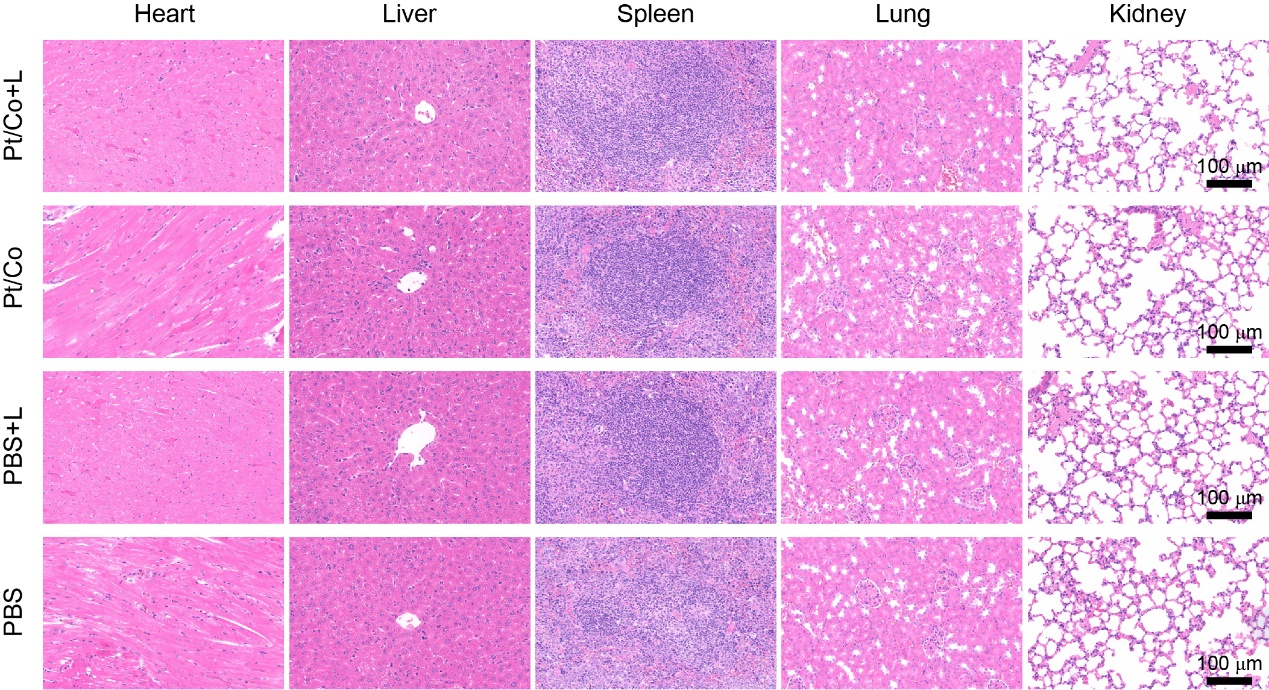


**Figure S40.** H&E stain of major organs taken from experimental mouse dealt with different treatments. Scale bar = 100 µm.


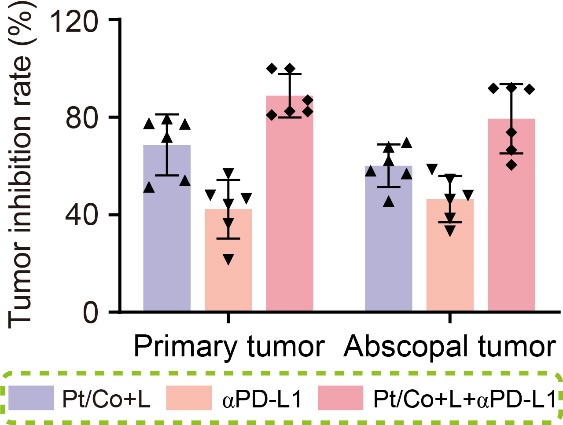


**Figure S41.** Tumor inhibition rates from two-origin tumor models.


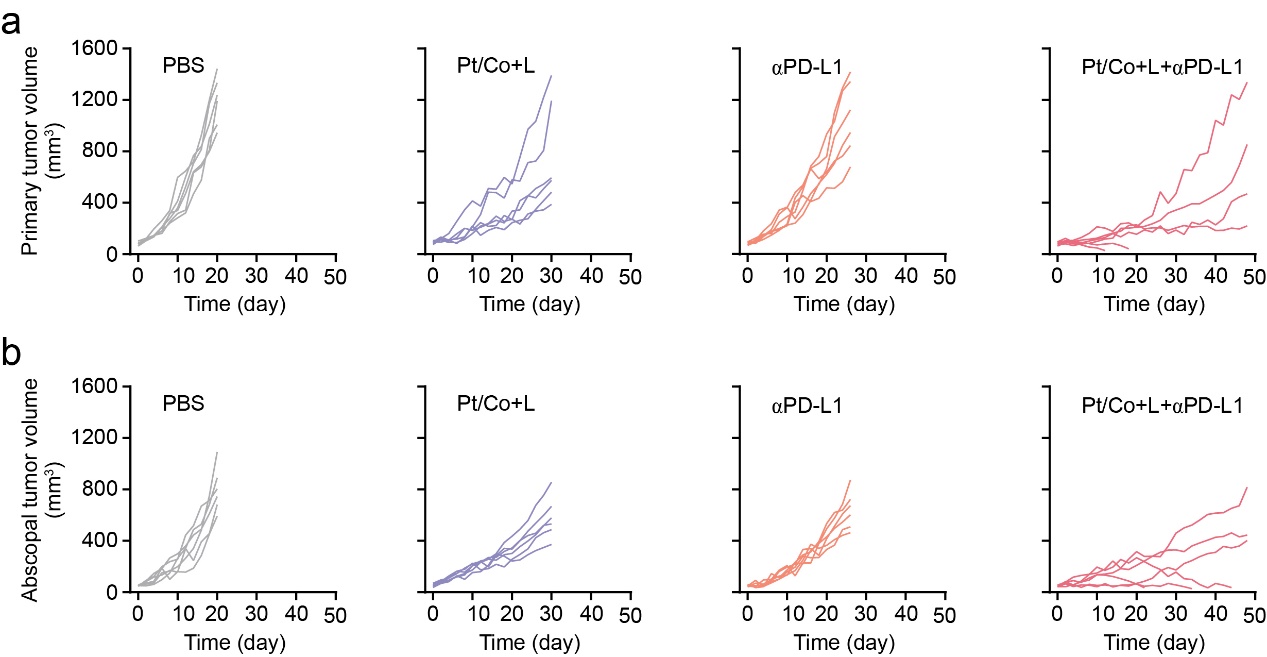


**Figure S42.** Tumor volume changes of (a) primary and (b) distal tumors with different treatments corresponding to Figure 6b and 6c.


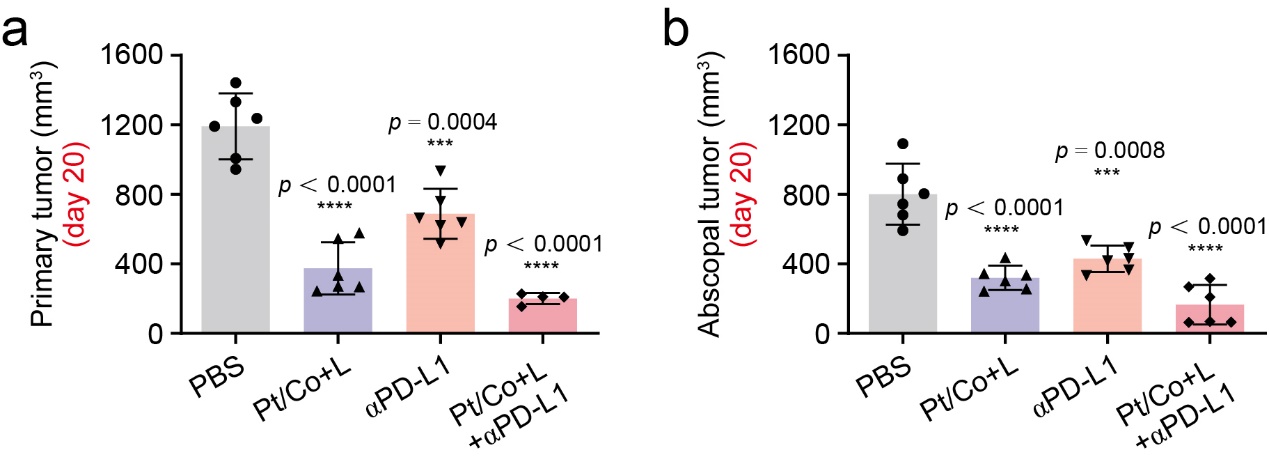


**Figure S43.** The volume of (a) primary and (b) abscopal tumors at 20^th^ day. The data are presented as mean ± SD (n = 6; ****p* < 0.001, *****p* < 0.0001).


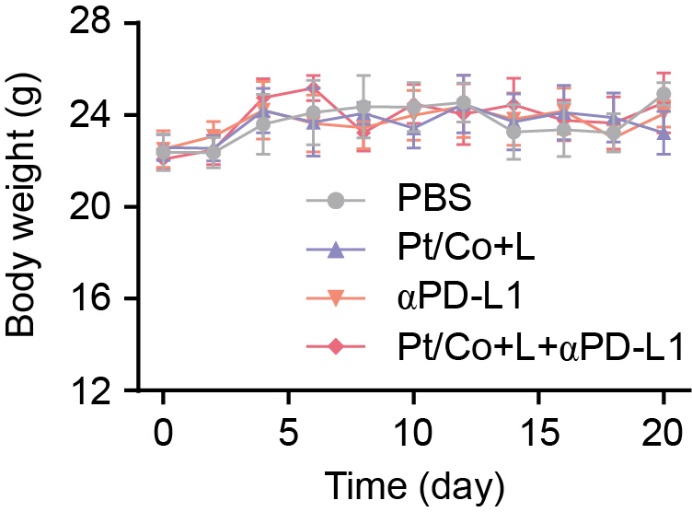


**Figure S44.** The body weight change of mice dealt with different treatments. The data are presented as mean ± SD (n = 6).


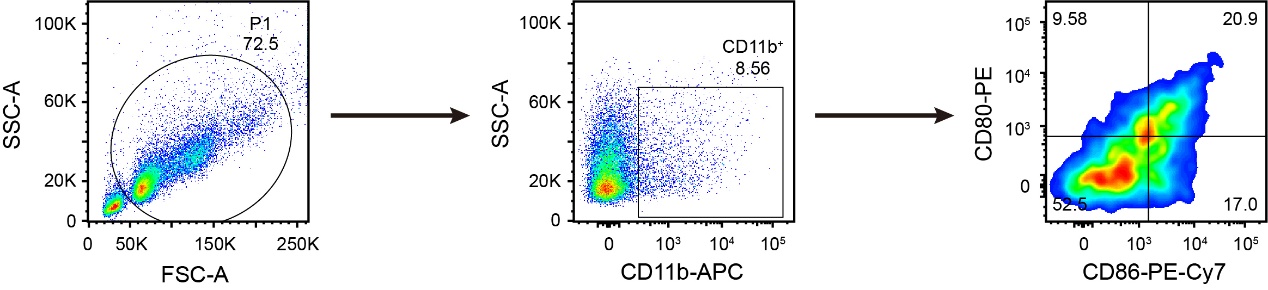


**Figure S45.** Gating strategy for analysis of mature DCs in the DLNs of primary tumor corresponding to Figure 6g.


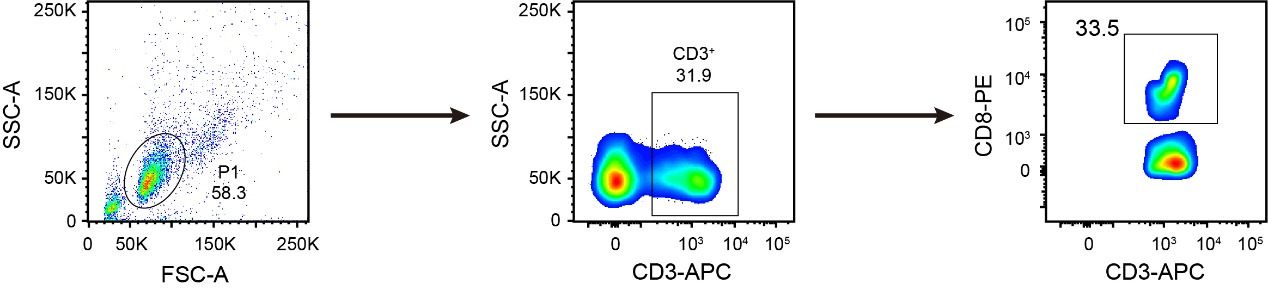


**Figure S46.** Gating strategy for analysis of CTLs in the spleen of mouse.


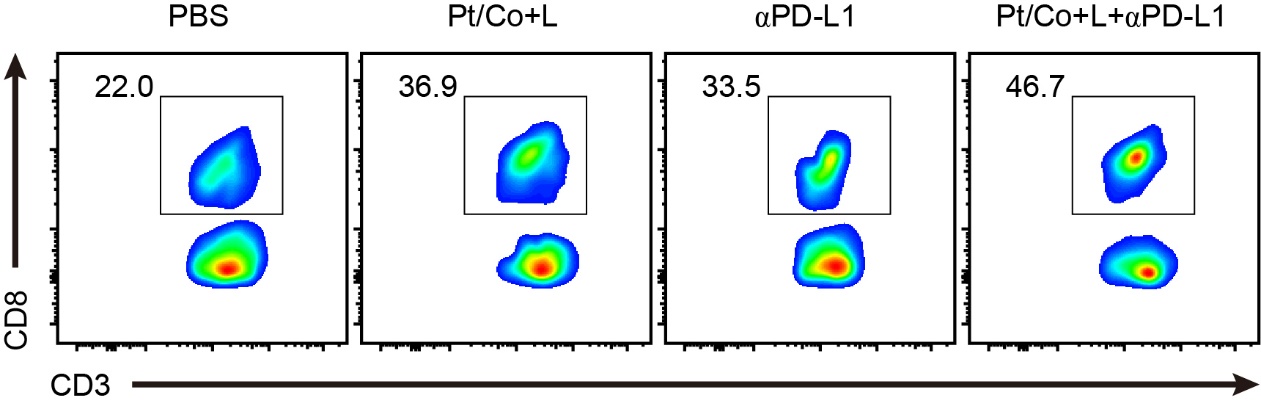


**Figure S47.** Flow cytometry of CTLs in the spleen of mouse corresponding to Figure 6i.


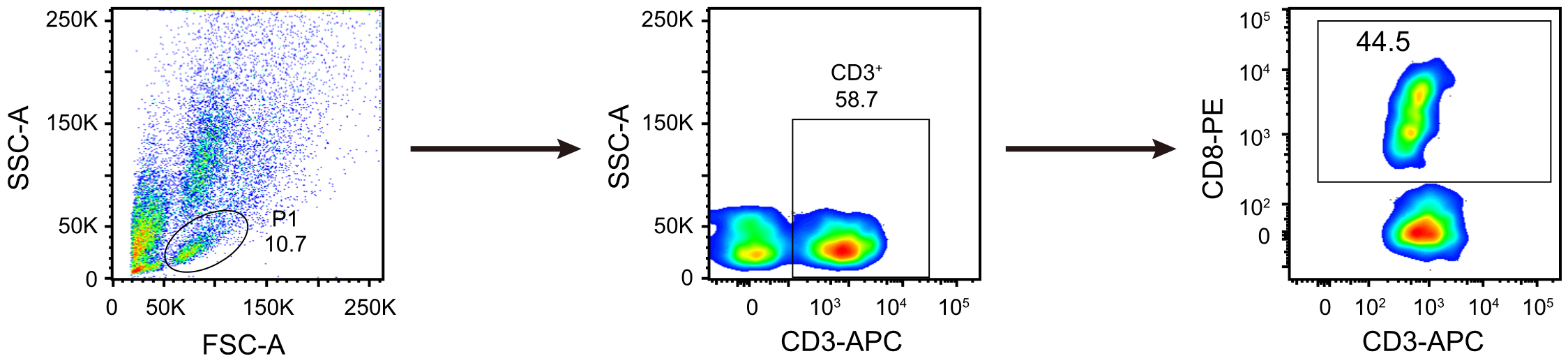


**Figure S48.** Gating strategy for analysis of CTLs in the abscopal tumor of mouse.


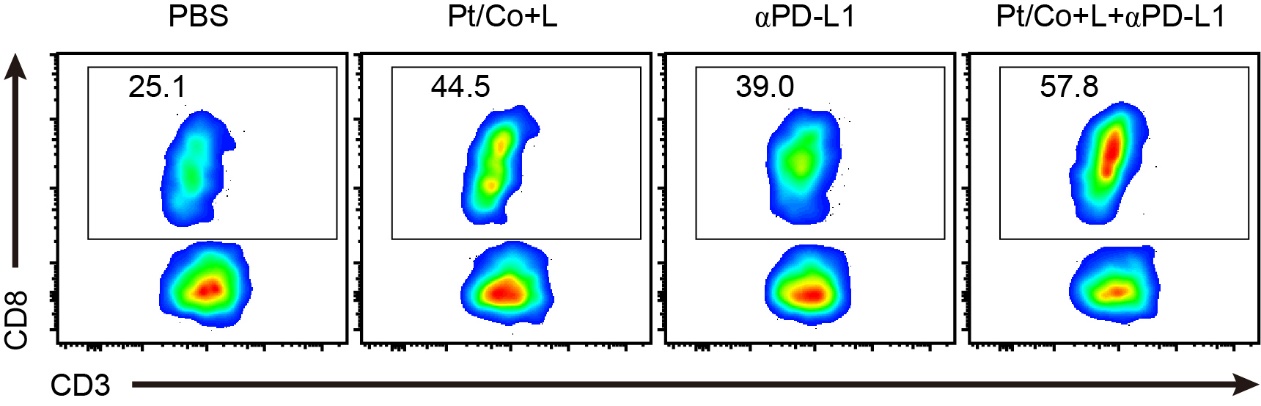


**Figure S49.** Flow cytometry of CTLs in the abscopal tumor of mouse corresponding to Figure 6j.


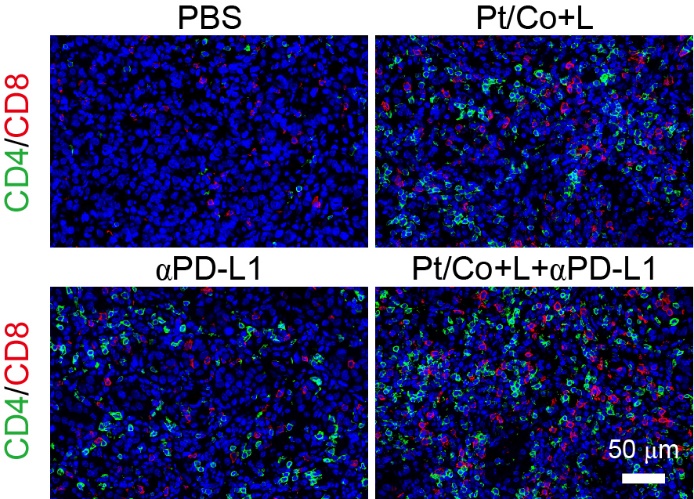


**Figure S50.** Immunofluorescence of CD4^+^/CD8^+^ T cells infiltrated in abscopal tumor of mouse. Scale bar = 50 µm.


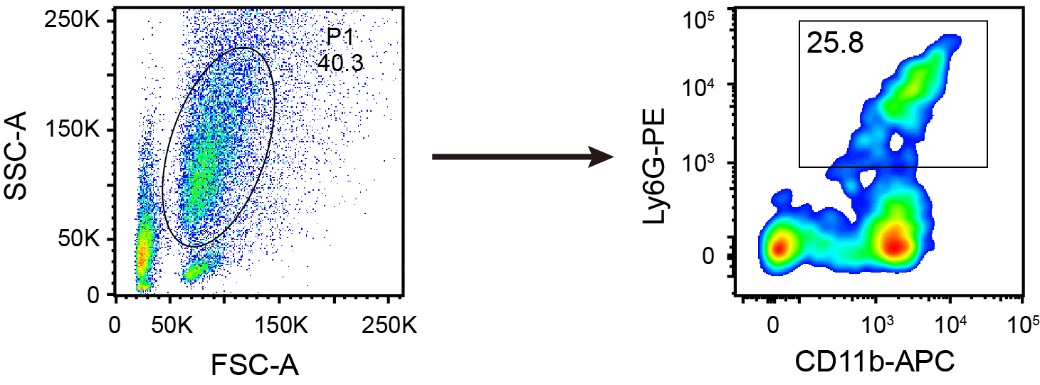


**Figure S51.** Gating strategy for analysis of MDSCs in the abscopal tumor of mouse.


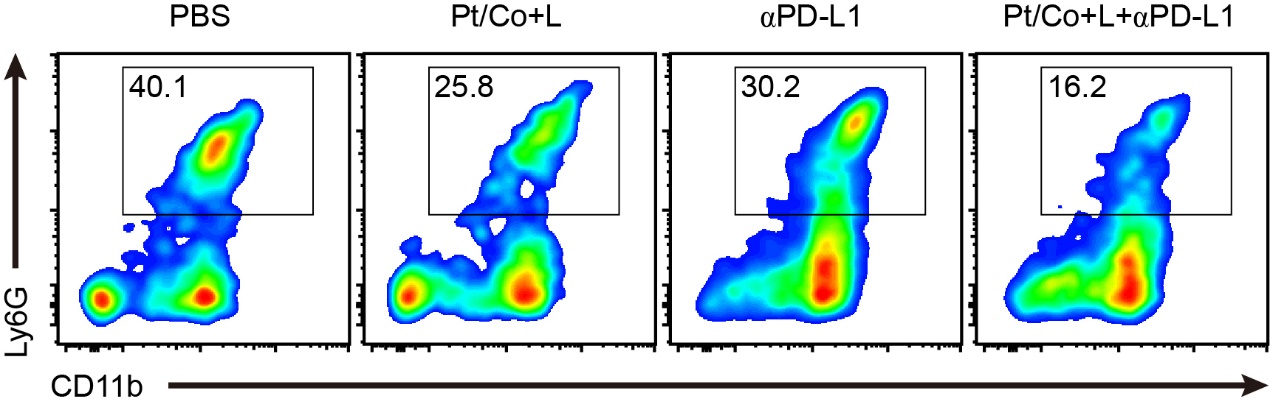


**Figure S52.** Flow cytometry of MDSCs in the abscopal tumor of mouse corresponding to Figure 6k.


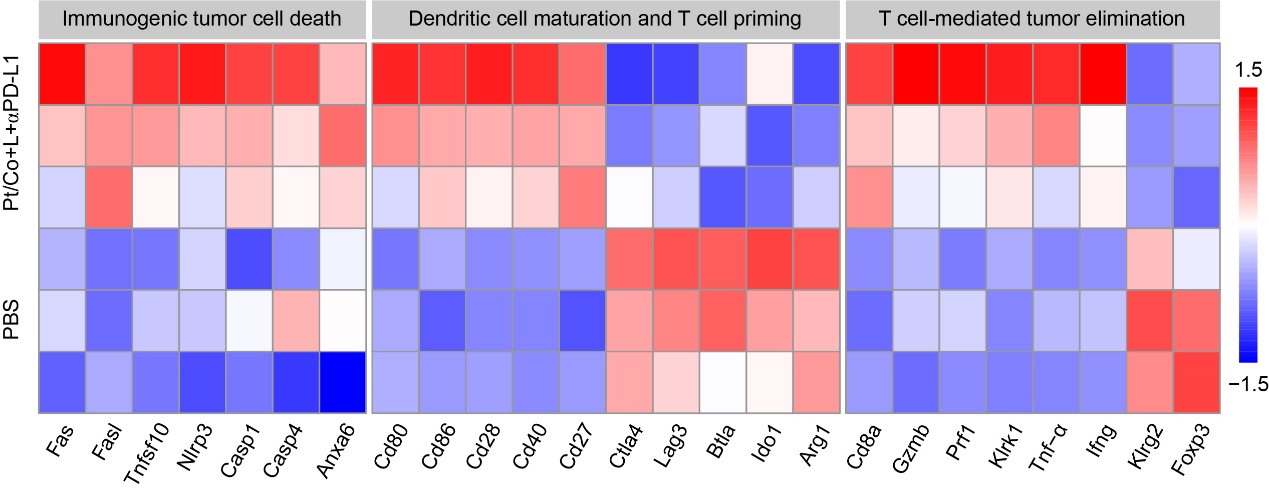


**Figure S53.** The heatmap analysis of interest DEGs classified by different immune function.


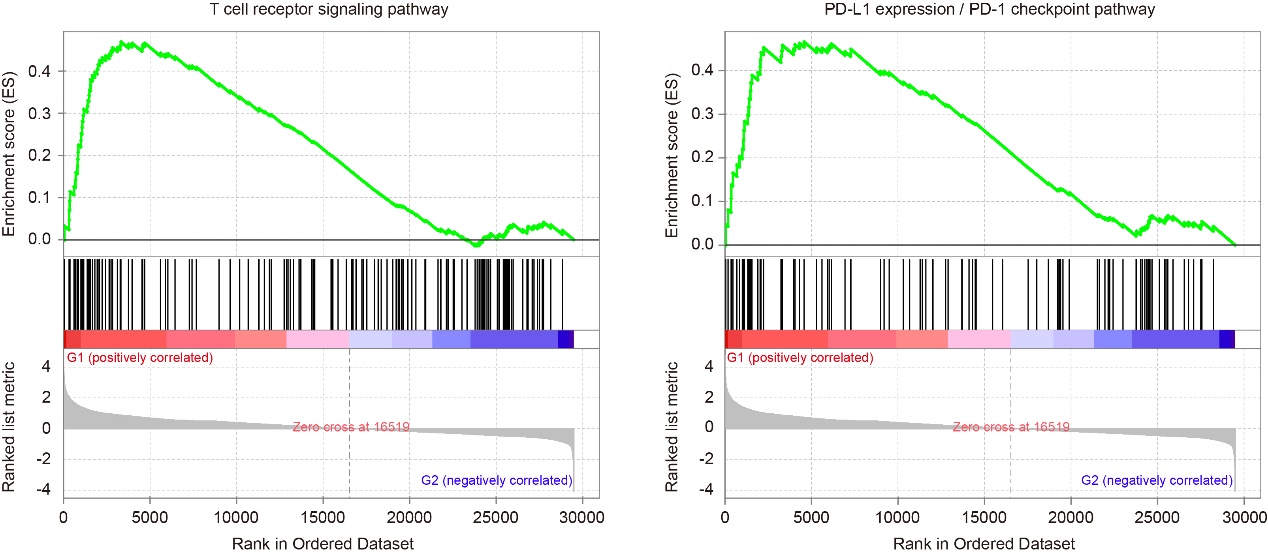


**Figure S54.** GSEA enrichment analysis of positively related immunological pathways in DEGs.


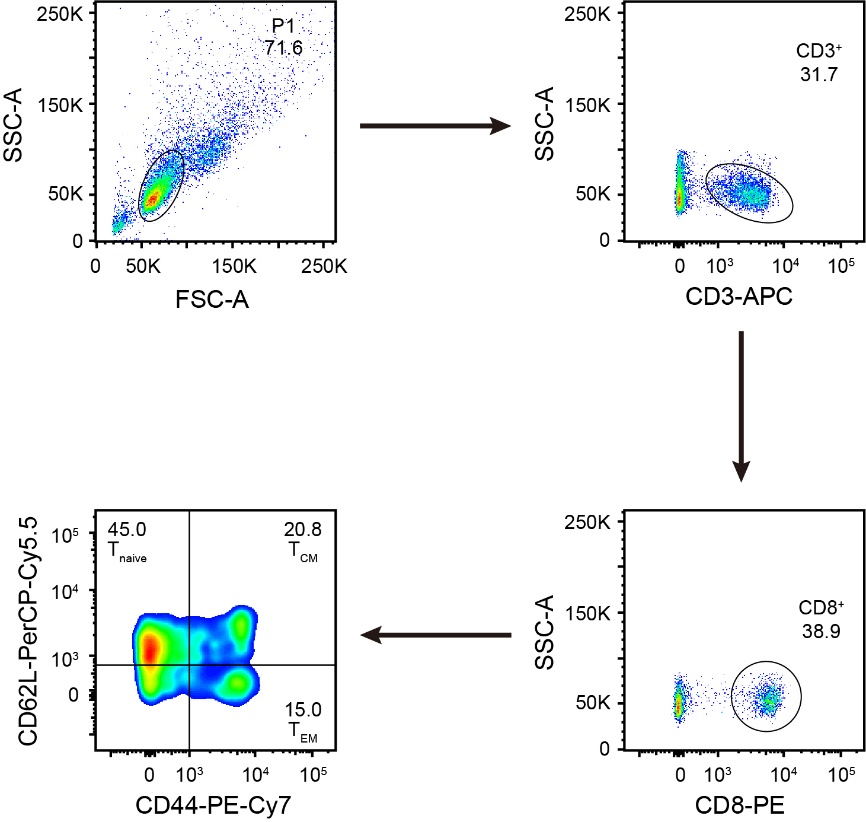


**Figure S55.** Gating strategy for analysis of memory T cells in CD8^+^ T cells.


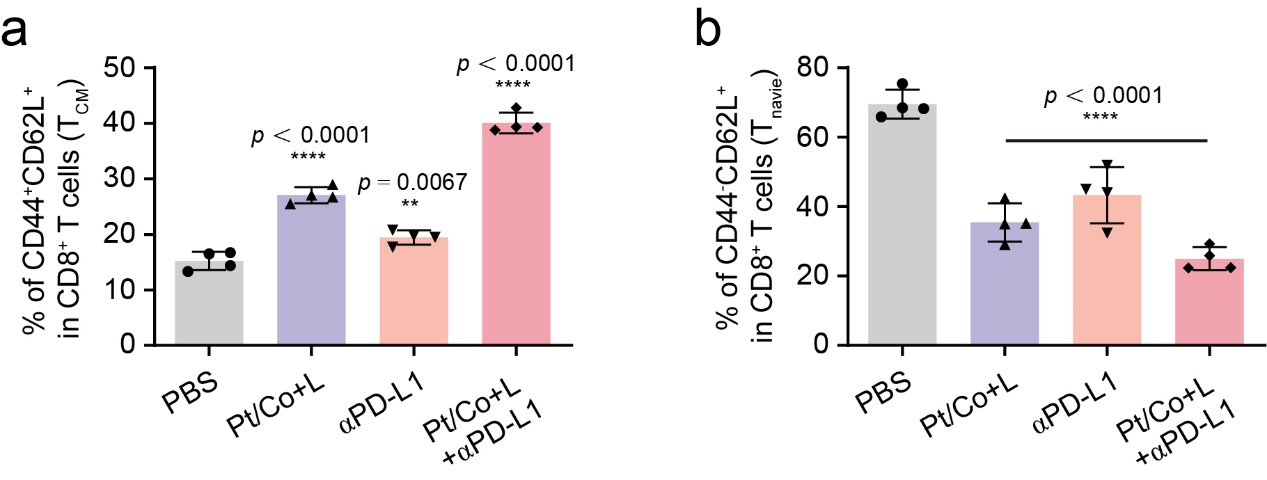


**Figure S56.** The ratio of (a) T_CM_ and (b) Tnaive in CD8^+^ T cells corresponding to Figure 8d. The data are presented as mean ± SD (n = 4; ***p* < 0.01, *****p* < 0.0001).


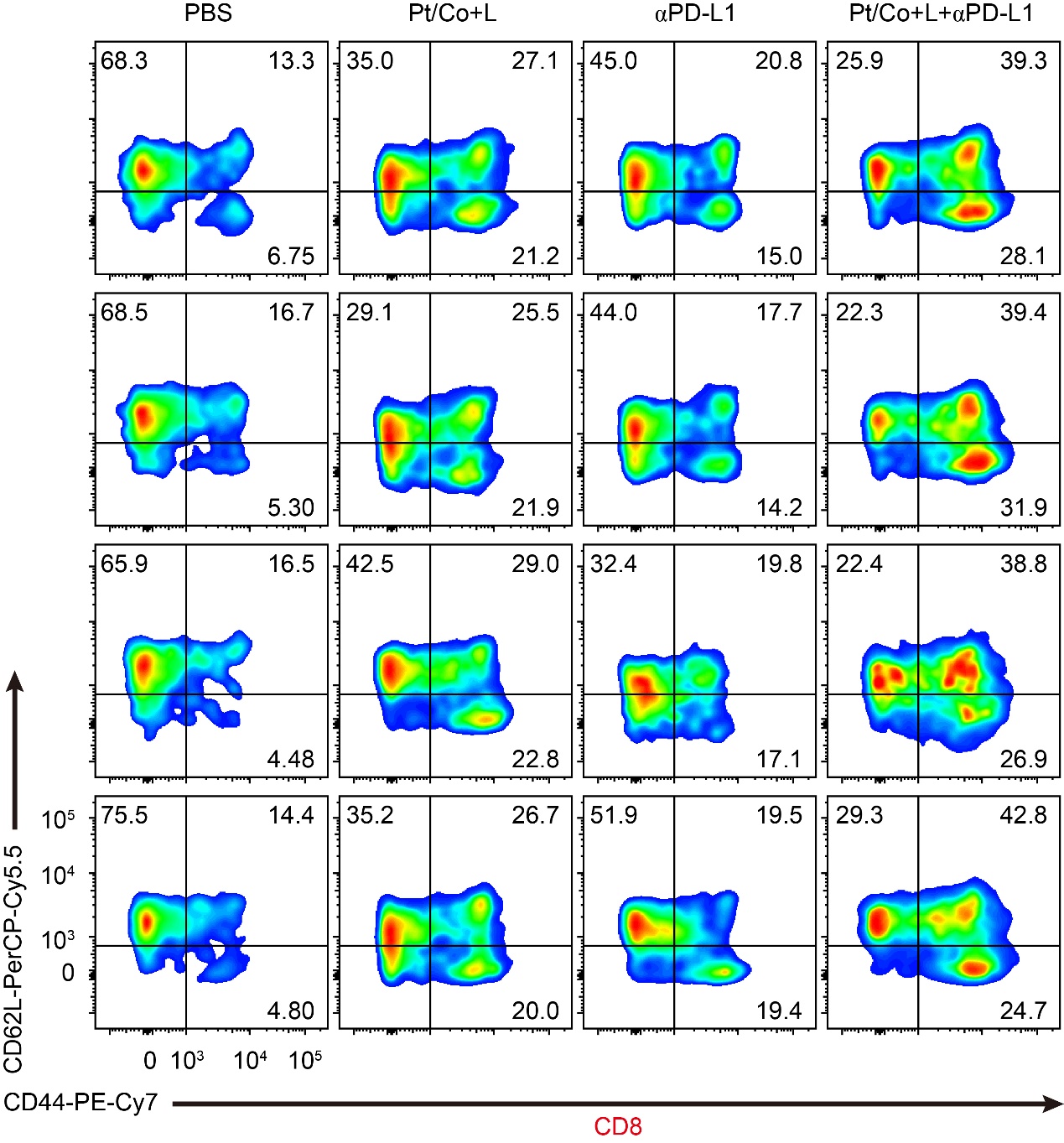


**Figure S57.** Flow cytometry of memory T cells in CD8^+^ T cells from all four parallel groups.


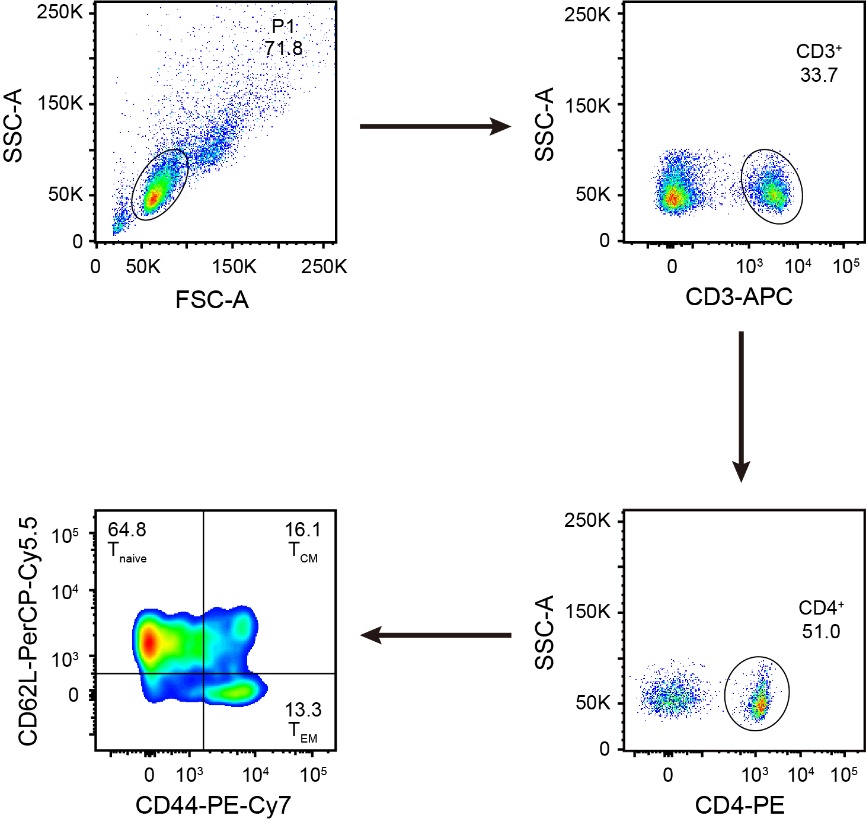


**Figure S58.** Gating strategy for analysis of memory T cells in CD4^+^ T cells.


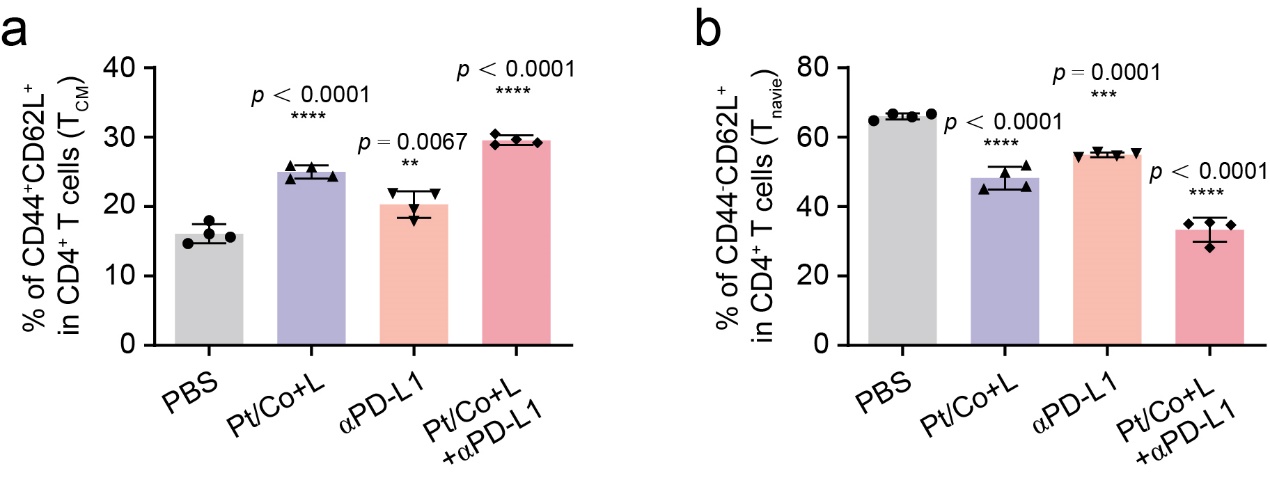


**Figure S59.** The ratio of (a) T_CM_ and (b) Tnaive in CD4^+^ T cells corresponding to Figure 6f. The data are presented as mean ± SD (n = 4; ***p* < 0.01, ****p* < 0.001, *****p* < 0.0001).


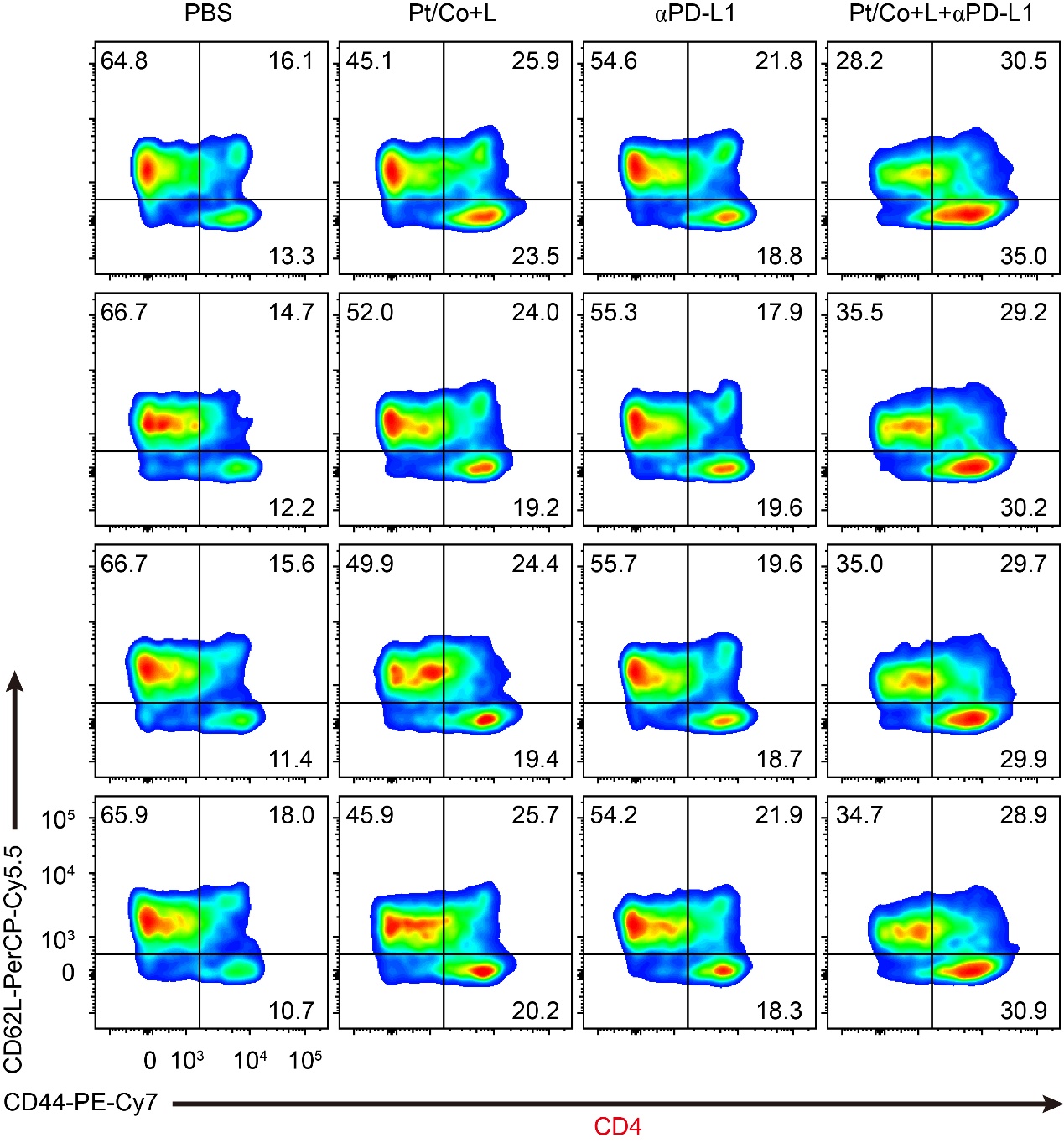


**Figure S60.** Flow cytometry of memory T cells in CD4^+^ T cells from all four parallel groups.
